# Supplementary material for: Developing undergraduate autism education for medical students: a qualitative study
Source: BMJ Paediatr Open. 2022 Aug 1;6(1):e001411. doi: 10.1136/bmjpo-2022-001411 (PMC9345079; doi:10.1136/bmjpo-2022-001411)
Supplement: Author's manuscript [file bmjpo-2022-001411.draft_revisions.pdf]

BMJ Paediatrics Open

BMJ Paediatrics Open is committed to open peer review. As part of this commitment we make the peer review history of every article we publish publicly available.

When an article is published we post the peer reviewers' comments and the authors' responses online. We also post the versions of the paper that were used during peer review. These are the versions that the peer review comments apply to.

The versions of the paper that follow are the versions that were submitted during the peer review process. They are not the versions of record or the final published versions. They should not be cited or distributed as the published version of this manuscript.

BMJ Paediatrics Open is an open access journal and the full, final, typeset and author-corrected version of record of the manuscript is available on our site with no access controls, subscription charges or pay-per-view fees (http://bmjpaedsopen.bmj.com).

If you have any questions on BMJ Paediatrics Open's open peer review process please email info.bmjpo@bmj.com

BMJ Paediatrics Open

# Developing undergraduate autism education for medical students: a qualitative study

| | |
|---|---|
| Journal: | *BMJ Paediatrics Open* |
| Manuscript ID | bmjpo-2022-001411 |
| Article Type: | Original research |
| Date Submitted by the Author: | 14-Feb-2022 |
| Complete List of Authors: | Dhuga, Yasmin; Brighton and Sussex Medical School<br>Feeney, Yvonne; Brighton and Sussex Medical School<br>Gallaher , Laura ; University of Sussex School of Media Arts and Humanities<br>White, Ann; Sussex Community NHS Foundation Trust<br>Wright , Juliet; Brighton and Sussex Medical School<br>Banerjee, Sube; University of Plymouth Faculty of Health and Human Sciences, Faculty of Health<br>Daley, Stephanie; Brighton and Sussex Medical School, |
| Keywords: | Health services research, Qualitative research |
| | |

SCHOLARONE™
Manuscripts

*I, the Submitting Author has the right to grant and does grant on behalf of all authors of the Work (as defined in the below author licence), an exclusive licence and/or a non-exclusive licence for contributions from authors who are: i) UK Crown employees; ii) where BMJ has agreed a CC-BY licence shall apply, and/or iii) in accordance with the terms applicable for US Federal Government officers or employees acting as part of their official duties; on a worldwide, perpetual, irrevocable, royalty-free basis to BMJ Publishing Group Ltd ("BMJ") its licensees and where the relevant Journal is co-owned by BMJ to the co-owners of the Journal, to publish the Work in this journal and any other BMJ products and to exploit all rights, as set out in our licence.*

*The Submitting Author accepts and understands that any supply made under these terms is made by BMJ to the Submitting Author unless you are acting as an employee on behalf of your employer or a postgraduate student of an affiliated institution which is paying any applicable article publishing charge ("APC") for Open Access articles. Where the Submitting Author wishes to make the Work available on an Open Access basis (and intends to pay the relevant APC), the terms of reuse of such Open Access shall be governed by a Creative Commons licence – details of these licences and which Creative Commons licence will apply to this Work are set out in our licence referred to above.*

*Other than as permitted in any relevant BMJ Author's Self Archiving Policies, I confirm this Work has not been accepted for publication elsewhere, is not being considered for publication elsewhere and does not duplicate material already published. I confirm all authors consent to publication of this Work and authorise the granting of this licence.*

Confidential: For Review Only

# Developing undergraduate autism education for medical students: a qualitative study

Yasmin Dhuga[a]

Yvonne Feeney[a]

Laura Gallaher[b]

Dr Ann White[c]

Professor Juliet Wright[a]

Professor Sube Banerjee[d]

Dr Stephanie Daley[a]

**Affiliations:**

a. Brighton and Sussex Medical School, UK
b. University of Sussex
c. Sussex Community NHS Foundation Trust, UK
d. Faculty of Health, University of Plymouth, UK

**Corresponding Author**

Dr Stephanie Daley

Address: Brighton & Sussex Medical School, Centre for Dementia Studies, Trafford Building, Room 101, University of Sussex, Falmer, BN1 9RY.

**Email:** s.daley@bsms.ac.uk **Tel:** 00447393 761947 **Twitter handle:** @stephdaleycds

**Word count: 2524**

Confidential: For Review Only

**Abstract**

**Background**:

Autistic people experience considerable health inequalities and have high rates of premature mortality, hospital admissions, and emergency department visits. This is in part due to a lack of autism awareness and poor understanding of the needs of autistic people. A new educational programme, Time for Autism, designed for medical students has been designed to address this challenge. This qualitative study was undertaken to support the development of this new programme, in order to a) understand the medical care experiences of parents of autistic children and b) assess their views on the acceptability of Time for Autism.

**Methods**:

A convenience sample of 11 parents of autistic children were recruited across South-East England. Semi-structured interviews were completed between October and December 2019. Interview transcripts were analysed using thematic analysis.

**Results:**

Three key themes were identified: Diagnosis; Experiences and expectations of medical care; and Time for Autism considerations. There was support for a dedicated autism education programme for medical students, and constructive feedback to inform and improve its delivery.

**Conclusion:** The findings from this study provide insights into the medical care experiences of parents/carers of autistic children. Understanding how parents/carers of autistic children would like medical care to be improved can be used to develop Time for Autism. Parental/carer support for the development of, and involvement in an autism medical education programme provides potential for the model to be replicated within other medical schools in the UK and internationally.

Confidential: For Review Only

*Keywords:* Autism, Medical Education, Qualitative, Healthcare experiences,

Confidential: For Review Only

## Background

Autism is a multi-faceted condition affecting neurodevelopment. It is it is characterised by impaired social interaction and communication, restricted interests, and repetitive, stereotypical behaviours (1). There are over 700,000 autistic people in the UK (2), with prevalence increasing.

Autistic people are more likely to have additional health conditions compared to the general population (3), such as anxiety-related disorders (4), sleep disorders (5), and gastrointestinal conditions (6). They are also at greater risk of health inequalities and are more likely to report negative healthcare experiences (7,8). Parents of autistic children describe the healthcare management of their children as inadequate (9). It is widely recognised that poor care experiences, as well as serious concerns about the quality of care provided to autistic people, demonstrates a need for improved understanding of autism across the health and social care workforce (10-13).

Medical doctors are rightly expected to have appropriate the necessary skills, competencies, and understanding to deliver high quality care to autistic people (14). In England, a competence framework for working with autistic people has been developed for the exsiting health and social care workforce(15).

However, at the undergraduate medical curriculum, a time when attitudes and openness to learning can be most malleable, autism teaching provision is often patchy and inconsistent. To enable the future generation of doctors to fully meet the needs of autistic people, and delivery care in a way which is person-centred, autism education needs to be routinely embedded within the undergraduate medical curricula. (10,16). There is a gap in the literature about the efficacy of successful interventions that can enhance student understanding of autism.

Confidential: For Review Only

While the literature on autism education for medical students is limited, there is evidence indicating that interventions incorporating lived experience can have a positive impact. One programme involving panel discussion with autistic people found improvements in knowledge and understanding in third year medial students (17). The use of a 'digistory' to share the experiences of parents/carers of autistic children was found to led to greater empathy, although significant change in attitude scores were not observed (18).

Incorporating lived experience to raise awareness and enhance skills towards autism in undergraduate medical students, the Time for Autism programme (TfA), was developed at Brighton and Sussex Medical School. Forming a mandatory component of the undergraduate medical curriculum, the programme was modelled on a similar educational programme (19) that successfully enhanced understanding and attitudes of dementia in large cohorts of healthcare students (20,21). In TfA, pairs of medical students visit a family (parent/carer with an autistic child) on three occasions over one academic year. Students learn about the lived experience of autism in the context of relational learning between the family and the student learns. The longitudinal nature allows students time to develop a fuller understanding of how autism affects the child and family over time, further facilitating better understanding of the condition.

A qualitative study was carried out with parent/carers of autistic children to further support the development of TfA in order to a) understand their experiences of medical care towards their autistic child and b) to understand their views about the acceptability, value and potential willingness to get involved in the TfA.

Confidential: For Review Only

## Methods

### Study design

We carried out a qualitative study using individual semi-structured interviews, in order to explore sensitive topics in more depth.

### Setting and participants

A convenience sample of participants were recruited. Eligible participants were parents/carers of an autistic child under the age of 18 years living in the South of England. Parents were recruited from local autism charities, local NHS trusts, and online parent forums and could self-refer to participate in the study. Participants were approached by email by the researchers and were not known to the research team before interview. Written, informed consent was obtained from all participants.

There was no patient or parent involvement in this study

### Procedure

Interviews were carried out by two female researchers, SD and LG. SD is an experienced qualitative post-doctoral researcher who trained and supervised LG. The majority of interviews took place at home with only the parent/carer present, at a time and venue suitable to participants, with the majority taking place at home. Interviews were undertaken on one occasion. Interviews were audio recorded and lasted between 30 and 60 minutes. A topic guide was reviewed by two autism clinicians to ensure suitability and interview questions focussed on the experience of the diagnosis of autism, experiences of consulting doctors about their autistic child, views about the Time for Autism programme, and willingness to participate. A pilot interview did not take place. Transcripts were not returned to participants, and feedback on findings was not sought.

Confidential: For Review Only

**Data analysis**

Interviews were transcribed verbatim and checked for accuracy. Transcripts were anonymised, and identifiable data was removed to maintain confidentiality. Thematic saturation was achieved after eleven interviews were completed, therefore data collection stopped at this point (22).

Transcripts were analysed using inductive thematic analysis (23). Familiarity with the data was achieved by reading the transcripts multiple times. To ensure rigour, two separate female researchers (YD and YF) independently coded two transcripts, before meeting to discuss the initial codes and resolve disagreements. The remaining transcripts were independently coded by the lead author; adding more codes when further meaningful data arose. The two researchers worked together to identify relationships between the codes and establish the main themes from the data. The themes were reviewed then refined and appropriate names were assigned to each theme. Microsoft Excel was used to aid the collation of the data.

To ensure that rigour and reflexivity, the researcher maintained fieldwork diaries and made notes after each interviews. The researchers met regularly to discuss their positionality and its impact on the findings.

**Ethical considerations**

This study was approved by South Central Hampshire B Research Ethics Committee (Ref 19/SC/0041).

**Results**

Confidential: For Review Only

Eleven participants took part in this study. Sociodemographic characteristics are shown in Table 1.

**Insert Table 1 here**

**Themes**

Three main themes were identified from the data: Diagnosis, Experiences and expectations of medical care, and Time for Autism considerations. These themes are summarised in table 2 and discussed below.

**Insert Table 2 here**

**Diagnosis**

This theme focused on the parental/carer experience of the doctor who diagnosed their child. The age of diagnosis ranged from 3 to 15 years.

Most participants identified some positive experiences of doctors who were perceived to be non-judgemental, and empathetic about the challenges they (parents/carers) faced prior to diagnosis. Being a good listener was a key quality valued by participants.

However, there were more negative, than positive experiences of doctors related to the diagnosis. Many participants felt that doctors lacked an appropriate level of awareness of autism, which for one parent had led to their children being misdiagnosed with other condition. Most participants reported their child was anxious, and that doctors would often attribute autistic traits as being associated with anxiety rather than autism. Another recurrent problem included doctors reported not using the correct diagnostic methods, for example not using standardised tools to enable them to screen for, or to diagnose autism, which delayed diagnosis further.

Confidential: For Review Only

## Experiences and expectations of medical care

Participants discussed the general experiences they encountered with doctors treating their autistic child. Two subthemes were identified: Experiences of medical care and Parental expectations of doctors.

*Experiences of medical care*

Participants valued doctors that took a holistic approach and demonstrated patience, allowing their autistic child time to adapt to new environments. Using personalised approaches and engaging with the child's interests before discussing their health issues was appreciated. Participants valued doctors that understood that autistic children have different needs, and those that took their time to listen and acknowledge parents/carers and their concerns.

Participants discussed many negative encounters. Most commonly, this related to a perceived lack of autism awareness and experience in doctors. Some participants described a perception of arrogance in doctors, and a failure to listen to parent/carer autism-related concerns about their child. As a result, participants suggested that doctors did not adjust their practice to me the needs of their child causing additional stress for the parent/carers. Some participants felt that doctors dismissed their concerns when their children become physically unwell, resulting use of A & E in crisis rather than primary care. Overall, participants concluded that doctors see autism as a problem.

*Parental expectations of doctors*

Participants expected that all doctors would hold understanding about autism and would have a willingness to involve parent/carers in decision making about their autistic child's care. Participants felt that doctors should involve parents/carers, early in the consultation so they can guide the doctor on how best to lead the consultation. Participants highlighted that as autism manifests differently, doctors should find out what works for each individual

Confidential: For Review Only

autistic child by asking the person who knows them best, their parent. They also suggested that doctors should think imaginatively when treating an autistic child, because they often present atypically with common conditions. Parents suggested that doctors should physically lower themselves to the child's level and speak calmly to them while giving them time to respond, using language that is easy to understand.

**Time for Autism considerations**

This theme focused on participant's views about the Time for Autism programme, and factors that would enhance their potential involvement. Two subthemes were identified: Positive attitudes about Time for Autism and Visit considerations.

*Positive attitudes about Time for Autism*

All participants expressed positive attitudes about TfA in being able to address the perceived lack of autism awareness in doctors. They felt that longitudinal contact with children in the community would enhance medical student understanding of autism and thereby improve their future practice. Participants suggested that gaining direct experience with autistic children would be more valuable than learning about autism form a textbook. They also suggested that students would be able to learn more about impact of autism on the family and therefore enable doctors to better manage difficult family situations. Finally, all of the participants reported their autistic children would enjoy being part of Time for Autism. Participants expressed hope that the programme would improve the medical experience for future parents/carers of autistic children and avoid similar difficulties.

*Visit considerations*

All participants were keen for TfA to take place, and provided a number of practical recommendations to the programme development. Students should avoid cancelling or

Confidential: For Review Only

rearranging visits as this could cause anxiety for the child. Participants suggested the need for flexibility during the visits based on the needs of the child. For example, some children might not participate in the conversation while others might enjoy engaging in an activity like drawing pictures. To reduce their child's anxiety, participants wanted to be able to prepare their child for the visits by receiving a small information sheet with a picture from the student. Participants were keen for students to observe the child in different environments because the child might exhibit different behaviours outside the home environment.

Confidential: For Review Only

**Discussion**

This study has generated novel insight on the medical experiences of families with autism. The results of this qualitative study can inform curriculum development of autism education, such as the Time for Autism programme (TfA).

Participants felt that doctors lack awareness around autism and wanted them to work in partnership with parents/carers when treating autistic children. They believed that tailored education could help raise awareness of autism and improve future treatment.

A delay of more than 4.5 years between parents detecting their children's developmental issues and receiving a formal autism diagnosis is common. The extent of this diagnostic delay is a predictor of overall parent satisfaction with the diagnostic experience (24), which was reflected in this study. Despite examples of positive experiences, participants overwhelmingly reported negative experiences relating to diagnosis; and perceived that a lack of autism awareness led to delays in diagnosis.

Providing parents/carers of autistic children with appropriate information concerning diagnosis and management is vital to ensure they can make informed decisions (25). The data is in this study makes clear that co-morbidities in autistic children are often misdiagnosed and mismanaged, with a lack of autism awareness leading to differing

perceptions between what the doctor thinks the parent/carer might need compared to the support which parents/carers feel they need (26- 28).

Mirroring the wider literature, participants felt they were not regarded as experts in dealing with their children's autism or valued when making joint decisions with medical personnel (29, 30). As demonstrated in these findings, such negative experiences can influence parents decisions when accessing health services for example only requesting medical help for acute illnesses and avoiding routine medical care for autistic-related problems (29, 30). These findings demonstrate that doctors must work in partnership with patients/carers of autistic children to improve treatment outcomes (27, 28, 31), as well as improved parent/carer satisfaction, improved family functioning (32-34).

The success of the doctor's interactions with autistic people depends on their ability to adapt their behaviour to address the needs of the person (35), unsurprisingly participants in this study valued doctors that personalised their approach when communicating with their autistic child. However, in order for doctors to adapt their behaviour, they need to be equipped with the necessary attitudes, skills, and knowledge to support autistic people (36). Participants believed that Time for Autism would improve student understanding of and approaches towards autistic people. This is supported by research which suggests that this kind of 'real life' exposure enables students to gain positive attitudes and values that cannot be learnt through textbooks (37). Longitudinal educational models which offer continuity of contact between student learners and patients/carers can enhance insight into the psychosocial side of caring for patients and understanding of chronic health conditions (38,39).

This study has three main limitations. First, participants were predominantly white, female parents/carers. This is of concern as it is suggested that autistic children from ethnic backgrounds experience more barriers in the healthcare setting (40), indicating further

Confidential: For Review Only

research on a more diverse sample of participants. Second, this study was undertaken in the south of England, and given the variation in support available geographically for autistic children (24), further research across a wider geographic area would be benefical.

A strength of this study is improved understanding of the experiences of autistic children and their parents/carers for the purposes of the design and delivery of autism education in undergraduate healthcare curricula.

To the best of our knowledge, this study is the first to explore the views of parents/carers of autistic children around the design of an autism medical educational programme. Their positive attitudes demonstrate enthusiasm for the inclusion of lived experience in educational delivery.

**Conclusion**

Despite calls for improved autism knowledge and awareness in the medical profession, this study found that parents of autistic children perceived a generalised lack of understanding of autism in medical settings.

While there is a lack of evidence demonstrating new and innovative ways to enhance autism awareness, education that incorporates lived experience can enhance positive attitudes and values towards chronic conditions. Findings from this study can be used in the design of initiatives to improve autism awareness in healthcare education. Participants in this study shared their enthusiasm for initiatives such as the Time for Autism programme to improve understanding of autism in medical education. Their insight can be used to enhance lived experience participation in similar longitudinal programmes.

Confidential: For Review Only

**What is already known on this topic**

- Parents/children of autistic children have high levels of dissatisfaction with the medical care provided to their children
- There is a lack of autism knowledge in doctors which contributes towards this problem
- For medical students, there is a lack of evidence as to how this knowledge gap can be addressed

**What this study adds**

- The identification of areas of practice in which parents/carers perceive that autism education should be addressed in medical students
- The willingness of parents/carers to contribute their lived experience within in autism education to address these concerns
- The introduction of a new autism education model, Time for Autism, for medical students in which lived experience is a core component

Confidential: For Review Only

**Acknowledgements**

We would like to thank the parents who took part in this study. We would also like to thank Alison Smith and Dr Lisa Butterworth-Salmon for reviewing the interview topic guides.

Confidential: For Review Only

## References


1. American Psychiatric Association. *Diagnostic and Statistical Manual of Mental Disorders. 5th ed. (DSM-5)*, 2013 Washington, DC: American Psychiatric Association.
2. National Autistic Society. *What is autism?* https://www.autism.org.uk/advice-and-guidance/what-is-autism [Accessed 3rd Dec 2020].
3. Saqr Y, Braun E, Porter K, Barnette D, Hanks C. Addressing medical needs of adolescents and adults with autism spectrum disorders in a primary care setting. *Autism.* 2017; *22*(1):51-61. doi:10.1177/1362361317709970
4. van Steensel FJ, Bögels SM, Perrin S. Anxiety disorders in children and adolescents with autistic spectrum disorders: a meta-analysis. *Clin Child Fam Psychol Rev.* 2011;14(3):302-317. doi:10.1007/s10567-011-0097-0
5. Kotagal S, Broomall E. Sleep in Children With Autism Spectrum Disorder. *Pediatric Neurology.* 2012; *47*(4):242-251. doi:https://doi.org/10.1016/j.pediatrneurol.2012.05.007
6. Chaidez V, Hansen RL, Hertz-Picciotto I. Gastrointestinal problems in children with autism, developmental delays or typical development. *J Autism Dev Disord*. 2014; *44*(5):1117-1127. doi:10.1007/s10803-013-1973-x.
7. Nicolaidis C, Raymaker D, McDonald K, Dern S, Boisclair WC, Ashkenazy E, Baggs A. Comparison of healthcare experiences in autistic and non-autistic adults: a cross-sectional online survey facilitated by an academic-community partnership. *J Gen Intern Med.* 2013; *28*(6):761-769. doi:10.1007/s11606-012-2262-7.
8. Liptak GS, Orlando M, Yingling JT, Theurer-Kaufman KL, Malay DP, Tompkins LA, Flynn JR. Satisfaction with primary health care received by families of children with


Confidential: For Review Only

developmental disabilities. *J Pediatr Health Care.* 2006; 20(4):245-252. doi:10.1016/j.pedhc.2005.12.008

9. Nik Adib NA, Ibrahim MI, Ab Rahman A, Bakar RS, Yahaya NA, Hussin S, Wan Mansor WNA. Predictors of Caregivers' Satisfaction with the Management of Children with Autism Spectrum Disorder: A Study at Multiple Levels of Health Care. *Int J Environ Res Public Health*. 2019; 16(10). doi:10.3390/ijerph16101684
10. Austriaco K, Aban I, Willig J, Kong M. Contemporary Trainee Knowledge of Autism: How Prepared Are Our Future Providers? *Front Pediatr.* 2019; *7*:165. doi:10.3389/fped.2019.00165
11. Bruder MB, Kerins G, Mazzarella C, Sims J, Stein N. Brief report: the medical care of adults with autism spectrum disorders: identifying the needs. *J Autism Dev Disord.* 2012;42(11):2498-2504. doi:10.1007/s10803-012-1496-x
12. Care Quality Commission. Out of sight – who cares?: Restraint, segregation and seclusion review. https://www.cqc.org.uk/sites/default/files/20201218_rssreview_report.pdf [Accessed 3rd Dec 2020].
13. Drum C, Krahn G, Peterson-Besse J, Horner-Johnson W, Newton K. Health of people with disabilities: Determinants and disparities. *Disability and Public Health*. 2009; 125-144.
14. Boyd K, Bridge E, McConnell M, Kates N, Stobbe, K. A Curriculum of Caring for People With Developmental Disabilities in Medical Education. *Journal on Developmental Disabilities.* 2019; 24(2):10-18.
15. Department of Health & Social Care. *Core Capabilities Framework for Supporting Autistic People.* [Internet] 2019. https://skillsforhealth.org.uk/wp-content/uploads/2020/11/Autism-Capabilities-Framework-Oct-2019.pdf. Accessed 2021 January 15].

Confidential: For Review Only

16. Ellias SD, Shah HR. A Study of Assessment of Knowledge of Childhood Autism among Medical Students in Mumbai. *Ann Indian Acad Neuro.* 2019; 22(2):164-169. doi:10.4103/aian.AIAN_486_17.

17. Havercamp SM, Ratliff-Schaub K, Macho PN, Johnson CN, Bush KL, Souders HT. Preparing tomorrow's doctors to care for patients with autism spectrum disorder. *Intellectual and Developmental Disabilities*. 2016; 54(3):202-216.

18. Codd A, Burford B, Petruso G, Davidson N, Vance G. Development and evaluation of a digistory about autistic spectrum disorder - a pilot study. *Educ Prim Care.* 2018;29(4):232-236. doi:10.1080/14739879.2018.1470906

19. Banerjee S, Farina N, Daley S, Grosvenor W, Hughes L, Hebditch M, Wright J. How do we enhance undergraduate healthcare education in dementia? A review of the role of innovative approaches and development of the Time for Dementia Programme. *Int J Geriatr Psychiatry.* 2016; *32*(1):68-75.

20. Banerjee S, Jones C, Wright J, Grosvenor W, Hebditch M, Hughes L, Daley S. A comparative study of the effect of the Time for Dementia programme on medical students. *Int J Geriatr Psychiatry.* 2021; 36(7):1011-1019. doi:10.1002/gps.5532

21. Daley S, Feeney Y, Grosvenor W, Hebditch M, Morley L, Sleater G, Banerjee S. A qualitative evaluation of the effect of a longitudinal dementia education programme on healthcare student knowledge and attitudes. *Age Ageing.* 2020; 49(6):1080-1086. doi:10.1093/ageing/afaa182

22. Saunders B, Sim J,Kingstone T, Baker S, Waterfield J, Bartlam B, Jinks C. Saturation in qualitative research: exploring its conceptualization and operationalization. *Qual Quant.* 2018; 52(4):1893-1907. doi:10.1007/s11135-017-0574-8

23. Braun V, Clarke V. Using thematic analysis in psychology. *Qualitative Research in Psychology*. 2006; 3(2):77-101. doi:10.1191/1478088706qp063oa

24. Crane L, Chester JW, Goddard L, Henry LA, Hill E. Experiences of autism diagnosis: A survey of over 1000 parents in the United Kingdom. *Autism.* 2016;20(2):153-62.

Confidential: For Review Only

25. Vahdat S, Hamzehgardeshi L, Hessam S, Hamzehgardeshi Z. Patient involvement in health care decision making: a review. *Iran Red Crescent Med J.* 2014; *16*(1):e12454. doi:10.5812/ircmj.12454

26. Liptak GS, Revell, GM. Community physician's role in case management of children with chronic illnesses. *Pediatrics.* 1989; *84*(3):465-471.

27. General Medical Council. *Domain 3: Communication partnership and teamwork.* https://www.gmc-uk.org/ethical-guidance/ethical-guidance-for-doctors/good-medical-practice/domain-3---communication-partnership-and-teamwork [Accessed 1st Jan 2021].

28. National Institue for Health and Care Excellence. *Autism.* https://www.nice.org.uk/guidance/qs51/chapter/quality-statement-3-personalised-plan [Accessed 14th Jan 2021].

29. Carbone PS, Behl DD, Azor V, Murphy NA. The medical home for children with autism spectrum disorders: parent and pediatrician perspectives. *J Autism Dev Disord.* 2010; 40(3):317-324. doi:10.1007/s10803-009-0874-5

30. Levy SE, Frasso R, Colantonio S, Reed H, Stein G, Barg FK, Fiks AG. Shared Decision Making and Treatment Decisions for Young Children With Autism Spectrum Disorder. *Acad Pediatr.* 2016; 16(6):571-578. doi:10.1016/j.acap.2016.04.007

31. Royal College of General Practiotioners. *Autism Spectrom Disorders Toolkit.* https://www.rcgp.org.uk/clinical-and-research/resources/toolkits/asd-toolkit.aspx [Accessed 13th Dec 2021].

32. Fiks AG, Localio AR, Alessandrini EA, Asch DA, Guevara JP. Shared decision-making in pediatrics: a national perspective. *Pediatrics.* 2010; 126(2):306-314. doi:10.1542/peds.2010-0526

33. Golnik A, Maccabee-Ryaboy N, Scal P, Wey A, Gaillard P. Shared decision making: improving care for children with autism. *Intellect Dev Disabil.* 2012; 50(4):322-331. doi:10.1352/1934-9556-50.4.322

Confidential: For Review Only

34. Kuhlthau KA, Bloom S, Van Cleave J, Knapp AA, Romm D, Klatka K, Perrin JM. Evidence for family-centered care for children with special health care needs: a systematic review. *Acad Pediatr.* 2011; 11(2):136-143. doi:10.1016/j.acap.2010.12.014

35. Nicolaidis C, Raymaker DM, Ashkenazy E, McDonald KE, Dern S, Baggs AE, Boisclair WC. "Respect the way I need to communicate with you": Healthcare experiences of adults on the autism spectrum. *Autism.* 2015;19(7):824-831. doi:10.1177/1362361315576221

36. Zuckerman KE, Lindly OJ, Bethell CD, Kuhlthau K. Family impacts among children with autism spectrum disorder: the role of health care quality. *Acad Pediatr.* 2014; 14(4):398-407. doi:10.1016/j.acap.2014.03.011

37. Evans DB, Henschen BL, Poncelet AN, Wilkerson L, Ogur B. Continuity in Undergraduate Medical Education: Mission Not Accomplished. *Journal of general internal medicine.* 2019; 34(10): 2254-2259. doi:10.1007/s11606-019-04949-0

38. Hirsh D, Gaufberg E, Ogur B, Cohen P, Krupat E, Cox M, Bor D. Educational outcomes of the Harvard Medical School-Cambridge integrated clerkship: a way forward for medical education. *Acad Med.* 2012; 87(5): 643-650. doi:10.1097/ACM.0b013e31824d9821

39. Ogrinc G, Mutha S, Irby DM. Evidence for longitudinal ambulatory care rotations: a review of the literature. *Acad Med.* 2002; 77(7): 688-693. doi:10.1097/00001888-200207000-00008

40. Magaña S, Parish SL, Rose RA, Timberlake M, Swaine JG. Racial and ethnic disparities in quality of health care among children with autism and other developmental disabilities. *Intellect Dev Disabil.* 2012;50(4): 287-299. doi:10.1352/1934-9556-50.4.287

**Table 1 Sociodemographic characteristics**

Confidential: For Review Only

| Baseline characteristic | n | % | Mean (SD) | Range (years) |
|---|---|---|---|---|
| **Age** | | | 43.64 (7.75) | 29-55 |
| **Age of autistic child.** | | | 10.18 (4.73) | 3-17 |
| **Ethnicity** | | | | |
| White background | 10 | 90.9 | | |
| Mixed background | 1 | 9.1 | | |
| **Relationship to child** | | | | |
| Mother | 10 | 90.9 | | |
| Father | 1 | 9.1 | | |
| **Marital status** | | | | |
| Married/partnered/ cohabiting | 11 | 100 | | |
| **Highest educational level** | | | | |
| No qualification | 1 | 9.1 | | |
| High school/ college | 6 | 54.5 | | |
| University or postgraduate degree | 4 | 36.4 | | |
| **Employment** | | | | |
| Unemployed | 1 | 9.1 | | |
| Employed | 10 | 90.9 | | |

Confidential: For Review Only

**Table 2: Participant quotations**

| Theme/Sub-theme | Quotation |
|---|---|
| **Diagnosis** | *"Dr X who is just amazing, she has been a paediatrician for many years, and she knows all about masking and everything…. So, we had the assessment, Dr X listened to what we were saying, and actually listened." (Participant 9)* |
| | *"This is our GP… "Well, he's not ripping up my surgery, so there can't be much wrong with him" and I just felt like putting my head in my hands, because I just thought … This is going to be a battle isn't it?" (Participant 1)* |
| | *"So, again they kind of felt …., it was just anxiety, it was kind of anxiety was the kind of prevailing sort of presentation and … they did the CBT approach." (Participant 7)* |
| **Experiences & expectations**<br><br>Experiences of medical care | *"…he sort of crouched down…., sort of put his elbows on the bed, and was like really interested in her.* (He) *didn't talk to us first, talked to her first, and then he said, "Do you mind if I just ask your mum and dad a couple of questions?" So, that was a really good example." (Participant 4)* |
| | *"…*[child] *wouldn't engage with the doctor, he wouldn't go, so, I rang the doctor, she said "Come and see me" without him* [child]*, to talk about him, which was great, because I was able to go. And she gave me as much time in the world to talk about him and his difficulties and what was going on at school, and she had the time and patience to sit there and listen to me and understand everything that was going on. And she said, we need to build up his tolerance to come in to visit the doctor. So, she said "Tell you what, next time, make an appointment with me… and if he won't get out the car, I'll come out and see him". (Participant 9)* |
| | *"the first GP that I saw … I asked at that time for …a referral psychology around his eating, I felt that they just kind of brushed that off and said that "Well, he's too young and it'll kind of sort itself out", but obviously we had several years of quite a restricted eating, he was seven of eight I thought that it was, you know, something more was going on. So, yeah, so it would have been really good if the GP had kind of spent a bit more time and taken me seriously." (Participant 7)* |
| Parental expectations of doctors | *"….are relentlessly dismissive and…I've had to take them to task recently, and ask for a referral to another team at a different hospital" (Participant 1)* |
| | *"I wouldn't want a doctor to think that they know one autistic child therefore we do this, necessarily, I mean to have some back up plans that's great, but I think really, I think what would surprise me and make me feel better, is if they were like "Okay, excellent! What's the best way for me to do this with her? Or I need to do this, these are the things I need to do, how's the best way to do it?" And then, you know, either I go "They all sound great," or "actually can we just do it like this". (Participant 2)* |
| **Time for Autism considerations**<br><br>Positive attitudes about TfA | *"I think it's brilliant … autism is something that is not well understood … I didn't realise how varied it is and how it displays itself really differently; my child is very different to the other children I know with autism, so, I think it's brilliant to have something that will help health professionals, or doctors in this case, understand a bit more…. So, I think it's great and I really support it." (Participant 2)* |
| | *"... some of the frustrations that we've experienced really, have been where people have had a very medicalised mechanist understanding of what autism is and … something that is a problem to be fixed, which is inappropriate I think from our experiences and also it's just offensive and quite upsetting and my kids get very upset when people put it that way. And so, I think one of the best ways I can think of for new doctors to get a different understanding of it is just to spend time around it, exactly as the programme is proposing. So, I think in that respect that's great." (Participant 6)* |

Confidential: For Review Only

| | |
|---|---|
| Visit considerations | *"... it would just have to make it like a home visit outside of school time, and don't be late. We can't be that flexible, once the time is booked, ideally it would be… fitting in with that family life would be best; after school would be best; and once you make it don't change it unless you have to, because that would then probably create some anxiety … but that would just be more because if we've done a lot of work around who's coming when…" (Participant 4)*<br><br>*"They* (students) *need to be able to make people feel comfortable if they're going to come into people's homes, because I think a lot of people, with kids with special needs, are invaded so frequently by social services and by all of these things; all these services that are set up to help can be very, very invasive..." (Participant 1)* |

Confidential: For Review Only

**Ethics approval and consent to participate**

This study was approved by South Central Hampshire B Research Ethics Committee (Ref 19/SC/0041). Informed written consent was obtained from participants included in the study.

**Consent for publication**

Not applicable

**Availability of data and materials**

The datasets used and/or analysed during the current study are available from the corresponding author on reasonable request

**Competing interests**

The authors declare there are no conflicts of interest.

**Funding**

This study was funded by Health Education England, Kent, Surrey, Sussex

The views expressed are those of the authors and not necessarily those of Health Education England, University of Sussex, University of Brighton or the Department of Health and Social Care

**Author Contributions:**

Conception and design: SD, AW, SB, JW

Data collection: SD, LG

Analysis: YD, YF, SD

Manuscript drafting: YD, SD, YF

All authors read and approved the final manuscript.

Confidential: For Review Only

**Acknowledgements**

We would like to thank the parents who took part in this study. We would also like to thank Alison Smith and Dr Lisa Butterworth-Salmon for reviewing the interview topic guides.

Confidential: For Review Only

BMJ Paediatrics Open

# Developing undergraduate autism education for medical students: a qualitative study

| | |
|---|---|
| Journal: | *BMJ Paediatrics Open* |
| Manuscript ID | bmjpo-2022-001411.R1 |
| Article Type: | Original research |
| Date Submitted by the Author: | 15-May-2022 |
| Complete List of Authors: | Dhuga, Yasmin; Brighton and Sussex Medical School<br>Feeney, Yvonne; Brighton and Sussex Medical School<br>Gallaher , Laura ; University of Sussex School of Media Arts and Humanities<br>White, Ann; Sussex Community NHS Foundation Trust<br>Wright , Juliet; Brighton and Sussex Medical School<br>Banerjee, Sube; University of Plymouth Faculty of Health and Human Sciences, Faculty of Health<br>Daley, Stephanie; Brighton and Sussex Medical School, |
| Keywords: | Health services research, Qualitative research |
| | |

SCHOLARONE™
Manuscripts

*I, the Submitting Author has the right to grant and does grant on behalf of all authors of the Work (as defined in the below author licence), an exclusive licence and/or a non-exclusive licence for contributions from authors who are: i) UK Crown employees; ii) where BMJ has agreed a CC-BY licence shall apply, and/or iii) in accordance with the terms applicable for US Federal Government officers or employees acting as part of their official duties; on a worldwide, perpetual, irrevocable, royalty-free basis to BMJ Publishing Group Ltd ("BMJ") its licensees and where the relevant Journal is co-owned by BMJ to the co-owners of the Journal, to publish the Work in this journal and any other BMJ products and to exploit all rights, as set out in our licence.*

*The Submitting Author accepts and understands that any supply made under these terms is made by BMJ to the Submitting Author unless you are acting as an employee on behalf of your employer or a postgraduate student of an affiliated institution which is paying any applicable article publishing charge ("APC") for Open Access articles. Where the Submitting Author wishes to make the Work available on an Open Access basis (and intends to pay the relevant APC), the terms of reuse of such Open Access shall be governed by a Creative Commons licence – details of these licences and which Creative Commons licence will apply to this Work are set out in our licence referred to above.*

*Other than as permitted in any relevant BMJ Author's Self Archiving Policies, I confirm this Work has not been accepted for publication elsewhere, is not being considered for publication elsewhere and does not duplicate material already published. I confirm all authors consent to publication of this Work and authorise the granting of this licence.*

Confidential: For Review Only

**Developing undergraduate autism education for medical students: a qualitative study**

Yasmin Dhuga[a]

Yvonne Feeney[a]

Laura Gallaher[b]

Dr Ann White[c]

Professor Juliet Wright[a]

Professor Sube Banerjee[d]

Dr Stephanie Daley[a]

**Affiliations:**

a. Brighton and Sussex Medical School, UK
b. University of Sussex
c. Sussex Community NHS Foundation Trust, UK
d. Faculty of Health, University of Plymouth, UK

**Corresponding Author**

Dr Stephanie Daley

Address: Brighton & Sussex Medical School, Centre for Dementia Studies, Trafford Building, Room 101, University of Sussex, Falmer, BN1 9RY.

**Email:** s.daley@bsms.ac.uk **Tel:** 00447393 761947 **Twitter handle:** @stephdaleycds

Confidential: For Review Only

**Word count: 2907**

**Abstract**

**Background**:

Autistic adults and children experience considerable health inequalities and have high rates of premature mortality, hospital admissions, and emergency department visits. This is in part due to a lack of autism awareness in the healthcare and social care workforce. A new educational programme, Time for Autism (TfA), for medical students is being developed to address this challenge. This qualitative study was undertaken to support the development of the new programme, in order to a) understand the medical care experiences of parents of autistic children and b) assess their views on the acceptability of the new TfA programme, and willingness to be involved.

**Methods**:

A convenience sample of 11 parents of autistic children were recruited across the South of England. The ages of the autistic children ranged from 3-17 years. Semi-structured interviews were completed between October and December 2019. Interview transcripts were analysed using thematic analysis.

**Results:**

Three key themes were identified: Diagnosis; Experiences of doctors and TfA considerations. There was support for, and willingness to take part in a dedicated autism education programme for medical students, and constructive feedback to inform and improve its delivery.

**Conclusion:** The findings from this study provide insights into the medical care experiences of parents/carers of autistic children. Understanding how parents/carers of autistic children would like medical care to be improved can be used to develop TfA and other autism programmes. Parental/carer support for the development of, and involvement in an autism medical education programme enhances the feasibility of the new programme.

Confidential: For Review Only

*Keywords:* Autism, Medical Education, Qualitative, Healthcare experiences,

Confidential: For Review Only

**Background**

Autism is a multi-faceted condition affecting neurodevelopment. It is characterised by impaired social interaction and communication, restricted interests, and repetitive, stereotypical behaviours (1). There are over 700,000 autistic people in the UK (2), with prevalence increasing (3). The cause of increasing prevalence is unknown but likely to be due in part to increased community awareness amongst the general population, including families and teachers and progress in case identification and definition (4).

Autistic people, both adults and children are more likely to have additional health conditions compared to the general population (5), including both mental health problems such as anxiety-related disorders (6), and sleep disorders (7), as well as physical health problems such as gastrointestinal conditions (8). Autistic adults and children are also at greater risk of health inequalities, and many autistic adults report negative healthcare experiences (9,10). Parents of autistic children describe the healthcare management of their children as inadequate (11). It is widely recognised that poor care experiences, as well as serious concerns about the quality of care provided to autistic adults and children demonstrates a need for improved understanding of autism across the lifespan within the health and social care workforce (12-16).

Medical doctors are expected to have appropriate skills, competencies, and understanding to deliver high quality care to autistic adults and children (14) In England, a competence framework for working with autistic people across the life span has been developed and embedded within statute for the exsiting health and social care workforce(17). However, at the undergraduate medical curriculum level, a time when attitudes and openness to learning can be most malleable, autism teaching provision is often patchy and inconsistent. To enable the future generation of doctors to fully meet the needs of autistic adults and children and deliver care in a way that is person-centred, autism education needs to be routinely embedded in the undergraduate medical curricula. (12,18). There is a gap in the literature

Confidential: For Review Only

about the efficacy of successful interventions that can enhance student understanding of autism.

While the literature on autism education for medical students is limited, there is some evidence indicating that interventions incorporating lived experience can have a positive impact. For example, one study reported an improvement in knowledge and understanding in third year medial students that completed panel discussions with autistic people (19). In another study, the use of a ‘digistory’ to share the experiences of parents/carers of autistic children led to medical students reporting greater empathy, improved confidence, and an ability to challenge negative stereotypes towards autism (20).

The incorporation of lived experience to raise awareness and enhance skills towards autism in undergraduate medical students is a key feature of a new autism education programme, that is being developed at Brighton and Sussex Medical School. The Time for Autism programme (TfA) will form a mandatory component of the undergraduate medical curriculum. The programme is modelled on a similar educational programme (22) that has successfully enhanced understanding and attitudes of dementia in large cohorts of healthcare students (22,23). In TfA, pairs of medical students will visit a family (parent/carer with an autistic child) on three occasions over one academic year. Students will learn about the lived experience of autism in the context of authentic relationship built between the family and student learners. It is intended that the longitudinal nature of the programme will allow students sufficient time to develop a fuller understanding of how autism affects the child and family over time, further facilitating improved understanding. Supporting teaching activities will focus on autism across the lifespan not just in childhood, and it is anticipated that several of the parents taking part in the new programme, will also have a diagnosis of autism, further enhancing the learning for students.

Confidential: For Review Only

Critical to the establishment of TfA will be the recruitment of volunteer families willing to have medical students visit them at home for the purpose of the new programme. To assess feasibility and inform the development of TfA, a qualitative study was carried out with parent/carers of autistic children to a) understand their experiences of medical care towards their autistic child and b) understand their views about the acceptability, value and potential willingness to get involved in the TfA.

**Methods**

**Study design**

We carried out a qualitative study using individual semi-structured interviews, to explore namely parent/carer views about the medical care experiences of their autistic children as well as a new medical education programme in more depth.

The study considered the *reality* of participants' perceptions through an exploration of their experiences and interpretations they attach to them, within a broader social *construct* (24). This epistemological approach loaned itself to the phenomenological methodology of thematic analysis and is steered by Braun and Clarke's guided approach to thematic analysis (25)

**Setting and participants**

A convenience sample of participants were recruited. Eligible participants were parents/carers of an autistic child under the age of 18 years living in the South of England. Participants were recruited from local autism charities, local NHS trusts, and online parent forums and they could self-refer to participate in the study. Participants were approached by email by the researchers and were not known to the research team before interview. Written, informed consent was obtained from all participants.

Confidential: For Review Only

Eleven participants took part in this study. Sociodemographic characteristics are shown in Table 1.

**Insert Table 1 here**

There was no specific lived experience advisory involvement in this study, in relation to the study design, delivery and analysis, although this has since been established for the TfA programme.

**Procedure**

Interviews were carried out by two female researchers, SD and LG. SD is an experienced qualitative post-doctoral researcher who trained and supervised LG. Each interview was completed at a time and venue suitable to participants; the majority took place at home with only the participant present. Interviews were undertaken on one occasion and were audio recorded and lasted between 30 and 60 minutes. A topic guide was reviewed by two autism clinicians to ensure suitability and interview questions focussed on the experience of the diagnosis of autism, experiences of consulting doctors (generally) about their autistic child, views about the TfA programme, and willingness to participate. A pilot interview did not take place. Transcripts were not returned to participants, and feedback on findings was not sought.

**Data analysis**

Interviews were transcribed verbatim and checked for accuracy. Transcripts were anonymised, and identifiable data was removed to maintain confidentiality. Thematic saturation was achieved after eleven interviews were completed, therefore data collection stopped at this point (26).

Confidential: For Review Only

Transcripts were analysed using inductive thematic analysis (25). Familiarity with the data was achieved by reading the transcripts multiple times. To ensure rigour, two separate female researchers (YD and YF) independently coded two transcripts, before meeting to discuss the initial codes and resolve disagreements. Fieldwork diaries were also reviewed. The remaining transcripts were independently coded by the lead author; adding more codes when further meaningful data arose. The two researchers worked together to identify relationships between the codes and establish the main themes from the data. The themes were reviewed then refined and appropriate names were assigned to each theme. Microsoft Excel was used to aid the collation of the data.

To enhance the analysis of data, the researchers maintained fieldwork diaries and made notes after each interview. In addressing reflexivity, the researchers met regularly to discuss their positionality and its impact on the findings during data collection and analysis.

**Ethical considerations**

This study was approved by South Central Hampshire B Research Ethics Committee (Ref 19/SC/0041).

**Results**

Three main themes were identified from the data: Diagnosis, Experiences of doctors, and TfA considerations. These themes are summarised in table 2 and discussed below.

**Insert Table 2 here**

Confidential: For Review Only

**Diagnosis**

Participants reported that the diagnosis of autism was usually provided by either the child's GP or their Paediatrician. The age of diagnosis ranged from 3 to 15 years. This theme captured both positive and negative experiences reported by the participants during the diagnosis of their child.

Positive experiences of diagnosis were influenced by doctors that were non-judgemental, and empathetic about the challenges which participants faced prior to diagnosis. Participants that felt listened to by medical professionals reported better experiences during the diagnosis of their child.

However, participants reported more negative than positive experiences of doctors relating to the diagnosis of autism in their child. The delay in obtaining a diagnosis, and the process of obtaining a diagnosis was often problematic for participants. Several explanations were provided to account for this; including a) a perceived lack of autism awareness b) a perception that parents' concerns were not being taken seriously, and c) perceived difficulty by GPs in identifying symptoms of autism. The presence of comorbid symptoms were perceived to contribute to the challenges obtaining a diagnosis, for example, anxiety was not always viewed as an autistic trait. Another recurrent problem reported was a belief that GP's did not always use the correct diagnostic methods, for example not using standardised tools to enable them to screen for, or to diagnose autism, which delayed diagnosis further.

**Experiences of doctors**

Participants discussed the general experiences they encountered with the wider range of doctors treating their autistic child.

Positive experience related to doctors who took a holistic approach and demonstrated patience, and allowed the autistic child time to adapt to new environments. Using

Confidential: For Review Only

personalised approaches and engaging with the child's interests before discussing their wider health issues was appreciated. Participants valued doctors who recognised that autistic children have different needs, and those that took their time to listen and acknowledge parents/carers and their concerns.

Equally, participants discussed many negative experiences of doctors. Most commonly, this related to a perceived lack of autism awareness and experience in doctors.

Some participants described a perceived arrogance in doctors, and a failure to listen to the parent/carer autism-related concerns about their child. As a result, participants suggested that doctors did not adjust their practice to meet the needs of their child causing additional stress for the parent/carers. Some participants felt that GP's in particular, dismissed their concerns when their children become physically unwell, resulting in avoidance of primary care by parents/carers and use of use of A & E as a first step in receiving medical care. Overall, participants concluded that doctors see autism as a 'problem'.

Participants expected that all doctors should have an appropriate understanding about autism and a willingness to involve parent/carers in decision making about their autistic child's care. Participants felt that doctors should involve parents/carers, early in the consultation so they can guide the doctor on how best to lead the consultation. Participants highlighted that as autism manifests differently, doctors should find out what works for each individual autistic child by asking the person who knows them best; their parent. Practical suggestions included doctors physically lowering themselves to the child's level and the use of easily understood language, rather than jargon.

Participants also suggested that doctors should be willing to deviate from routine established practice when assessing an autistic child, because they may present atypically

Confidential: For Review Only

or the child may be unwilling to engage, for example be willing to assess the child in the family car if the child won't come into the GP practice.

**TfA considerations**

This theme focused on participant's views about the TfA programme, and potential willingness to take part. Two subthemes were identified: Positive attitudes about TfA and Visit considerations.

*Positive attitudes about TfA*

All participants were positive about the development of the TfA programme and the potential for it to address the perceived lack of autism awareness in doctors. Participants stated that longitudinal contact with children in the community would enhance medical student understanding of autism and thereby improve their future practice. Participants suggested that gaining direct experience with autistic children would be more valuable than learning about autism from a textbook. Participants also suggested that medical students would be able to learn more about the impact of autism on the family; potentially enabling doctors to better manage difficult family situations. Finally, all participants reported their autistic children would also enjoy being part of TfA.

*Visit considerations*

All participants were keen for TfA to take place and provided practical recommendations to support programme development. Participants advised that students should avoid cancelling or rearranging visits as this could cause anxiety for the child. Participants suggested the need for flexibility during the visits based on the needs of the child, for example, some children might not participate in the conversation while others might enjoy engaging in an activity like drawing pictures. It was perceived that advance information and a photograph of the medical student would reduce possible anxiety in the autistic child.

Confidential: For Review Only

**Discussion**

This study has generated insights on the medical experiences of families with autism. The results of this qualitative study will be used to inform the development of the TfA programme and can be used to inform other such programmes.

Whilst some of the direct examples provided by participants related to GP's and Paediatricians, participants perceived that most doctors lack awareness of autism and did not work in partnership with parents of autistic children. Participants were strongly supportive of tailored education to address these concerns. Crucially for the development of TfA, participants in this study reported a willingness to take part directly in medical education and share their lived experience to address this challenge. This suggests that the TfA programme is a feasible aspiration.

Participants in in this study highlighted delays in obtaining an autism diagnosis for their child and frustration with the process. A delay of more than 4.5 years between parents detecting their children's developmental issues and receiving a formal autism diagnosis is reported in the literature, and the extent of this diagnostic delay is a predictor of overall parent satisfaction with the diagnostic experience (27). Despite examples of positive experiences, participants overwhelmingly reported negative experiences relating to diagnosis; and perceived that a lack of autism awareness led to delays in diagnosis.

Participants in this study identified that medical management of co-morbidities was often problematic for both parents/carers and the autistic children, leading to differing perceptions of health needs and avoidance of primary care by some participants. This is supported in the wider literature, where it is recognised that co-morbidities in autistic children are often misdiagnosed and mismanaged (28- 30).

Confidential: For Review Only

Mirroring the wider literature, participants felt they were not regarded as experts in dealing with their children's autism or valued when making joint decisions with doctors (31, 32). These findings demonstrate that doctors must work in partnership with patients/carers of autistic children to improve treatment outcomes (29, 30, 33), as well as improved parent/carer satisfaction.

The success of the doctor's interactions with autistic people depends on their ability to adapt their behaviour to address the needs of the person (37). Unsurprisingly participants in this study valued doctors personalising their approach when communicating with autistic children. However, in order for doctors to adapt their behaviour, they need to be equipped with the necessary attitudes, skills, and knowledge to support autistic people (38).

Participants perceived that TfA would improve student understanding of and approaches towards autistic people. This is supported by research which suggests that this kind of 'real life' exposure enables students to gain positive attitudes and values that cannot be learnt through textbooks (39). Longitudinal educational models that offer continuity of contact between student learners and patients/carers can enhance insight into the psychosocial side of caring for patients and understanding of chronic health conditions (40,41).

This study has three main limitations. First, this study used a convenience sample, and therefore lacks diversity in terms of ethnicity and economic status. Our participants were predominantly white, female parents/carers. We were therefore not able to assess the experiences of all groups of carers, which is of concern as it is suggested that autistic children from ethnic backgrounds experience more barriers in the healthcare setting (42). Additionally, it is not clear whether parents from more diverse backgrounds would be willing to take part in a programme like TfA, potentially limiting exposure to the full range of experiences for our medical students. It is evident that further research using a more diverse sample of participants is necessary. Second, this study was undertaken in the south of

Confidential: For Review Only

England, and given the variation in support available geographically for autistic children (27), further research across a wider geographic area would be beneficial. Finally, lived experience involvement of parents/carers to advise on the design, delivery and analysis would have strengthened the study.

A strength of this study is an improved understanding of the medical care experiences of autistic children and their parents/carers for the purposes of the design and delivery of autism education in undergraduate healthcare curricula. Their positive attitudes towards TfA demonstrates enthusiasm for the active involvement of parents/carers and autistic children in educational delivery.

Confidential: For Review Only

**Conclusion**

Despite calls for improved autism knowledge and awareness in the medical profession, this study found that parents/carers of autistic children perceived a generalised lack of understanding of autism in medical settings.

While there is a lack of evidence demonstrating new and innovative ways to enhance autism awareness, education that incorporates lived experience can enhance positive attitudes and values towards chronic conditions. Findings from this study can be used in the design of new initiatives to improve autism awareness in healthcare education. Participants in this study were willing to take part in new programmes, such as TfA to improve understanding of autism in future doctors. A full evaluation of the experience of medical students and parents/carers in TfA will be undertaken on the new programme.

**What is already known on this topic**

- Parents/children of autistic children have high levels of dissatisfaction with the medical care provided to their children
- There is a lack of autism knowledge in doctors which contributes towards this problem
- For medical students, there is a lack of evidence as to how this knowledge gap can be addressed

**What this study adds**

- The identification of areas of practice in which parents/carers perceive that autism education should be addressed in medical students
- The willingness of parents/carers to contribute their lived experience within in autism education to address these concerns
- The introduction of a new autism education model, Time for Autism, for medical students in which lived experience is a core component

**Acknowledgements**

We would like to thank the parents who took part in this study. We would also like to thank Alison Smith and Dr Lisa Butterworth-Salmon for reviewing the interview topic guides.

Confidential: For Review Only

Confidential: For Review Only

**References**


1. American Psychiatric Association. *Diagnostic and Statistical Manual of Mental Disorders. 5th ed. (DSM-5)*, 2013 Washington, DC: American Psychiatric Association.
2. National Autistic Society. *What is autism?* https://www.autism.org.uk/advice-and-guidance/what-is-autism [Accessed 3rd Dec 2020].
3. King M, Bearman P, Diagnostic change and the increased prevalence of autism. *International Journal of Epidemiology*. 2009; 38(5): 1224–1234. doi.org/10.1093/ije/dyp261
4. Zeidan J, Fombonne E, Scorah J, Ibrahim A, Durkin MS, Saxena S, Yusuf A, Shih A, & Elsabbagh, M. Global prevalence of autism: A systematic review update. *Autism Research*. 2022; 1– 13. doi.org/10.1002/aur.2696
5. Saqr Y, Braun E, Porter K, Barnette D, Hanks C. Addressing medical needs of adolescents and adults with autism spectrum disorders in a primary care setting. *Autism.* 2017; *22*(1):51-61. doi:10.1177/1362361317709970
6. van Steensel FJ, Bögels SM, Perrin S. Anxiety disorders in children and adolescents with autistic spectrum disorders: a meta-analysis. *Clin Child Fam Psychol Rev.* 2011;14(3):302-317. doi:10.1007/s10567-011-0097-0

7. Kotagal S, Broomall E. Sleep in Children With Autism Spectrum Disorder. *Pediatric Neurology.* 2012; *47*(4):242-251. doi:https://doi.org/10.1016/j.pediatrneurol.2012.05.007
8. Chaidez V, Hansen RL, Hertz-Picciotto I. Gastrointestinal problems in children with autism, developmental delays or typical development. *J Autism Dev Disord*. 2014; *44*(5):1117-1127. doi:10.1007/s10803-013-1973-x.
9. Nicolaidis C, Raymaker D, McDonald K, Dern S, Boisclair WC, Ashkenazy E, Baggs A. Comparison of healthcare experiences in autistic and non-autistic adults: a cross-sectional online survey facilitated by an academic-community partnership. *J Gen Intern Med.* 2013; *28*(6):761-769. doi:10.1007/s11606-012-2262-7.
10. Liptak GS, Orlando M, Yingling JT, Theurer-Kaufman KL, Malay DP, Tompkins LA, Flynn JR. Satisfaction with primary health care received by families of children with developmental disabilities. *J Pediatr Health Care.* 2006; 20(4):245-252. doi:10.1016/j.pedhc.2005.12.008
11. Nik Adib NA, Ibrahim MI, Ab Rahman A, Bakar RS, Yahaya NA, Hussin S, Wan Mansor WNA. Predictors of Caregivers' Satisfaction with the Management of Children with Autism Spectrum Disorder: A Study at Multiple Levels of Health Care. *Int J Environ Res Public Health*. 2019; 16(10). doi:10.3390/ijerph16101684
12. Austriaco K, Aban I, Willig J, Kong M. Contemporary Trainee Knowledge of Autism: How Prepared Are Our Future Providers? *Front Pediatr.* 2019; *7*:165. doi:10.3389/fped.2019.00165
13. Bruder MB, Kerins G, Mazzarella C, Sims J, Stein N. Brief report: the medical care of adults with autism spectrum disorders: identifying the needs. *J Autism Dev Disord.* 2012;42(11):2498-2504. doi:10.1007/s10803-012-1496-x
14. Care Quality Commission. Out of sight – who cares?: Restraint, segregation and seclusion review. https://www.cqc.org.uk/sites/default/files/20201218_rssreview_report.pdf [Accessed 3rd Dec 2020].

Confidential: For Review Only

15. Drum C, Krahn G, Peterson-Besse J, Horner-Johnson W, Newton K. Health of people with disabilities: Determinants and disparities. *Disability and Public Health*. 2009; 125-144.

16. World Health Organisation, 2022, Autism, World Health Organisation- Geneva (report). https://www.who.int/news-room/fact-sheets/detail/autism-spectrum-disorders

17. Department of Health & Social Care. *Core Capabilities Framework for Supporting Autistic People.* [Internet] 2019. https://skillsforhealth.org.uk/wp-content/uploads/2020/11/Autism-Capabilities-Framework-Oct-2019.pdf. Accessed 2021 January 15].

18. Ellias SD, Shah HR. A Study of Assessment of Knowledge of Childhood Autism among Medical Students in Mumbai. *Ann Indian Acad Neuro.* 2019; 22(2):164-169. doi:10.4103/aian.AIAN_486_17.

19. Havercamp SM, Ratliff-Schaub K, Macho PN, Johnson CN, Bush KL, Souders HT. Preparing tomorrow's doctors to care for patients with autism spectrum disorder. *Intellectual and Developmental Disabilities*. 2016; 54(3):202-216.

20. Codd A, Burford B, Petruso G, Davidson N, Vance G. Development and evaluation of a digistory about autistic spectrum disorder - a pilot study. *Educ Prim Care.* 2018;29(4):232-236. doi:10.1080/14739879.2018.1470906

21. Banerjee S, Farina N, Daley S, Grosvenor W, Hughes L, Hebditch M, Wright J. How do we enhance undergraduate healthcare education in dementia? A review of the role of innovative approaches and development of the Time for Dementia Programme. *Int J Geriatr Psychiatry.* 2016; *32*(1):68-75.

22. Banerjee S, Jones C, Wright J, Grosvenor W, Hebditch M, Hughes L, Daley S. A comparative study of the effect of the Time for Dementia programme on medical students. *Int J Geriatr Psychiatry.* 2021; 36(7):1011-1019. doi:10.1002/gps.5532

23. Daley S, Feeney Y, Grosvenor W, Hebditch M, Morley L, Sleater G, Banerjee S. A qualitative evaluation of the effect of a longitudinal dementia education programme

Confidential: For Review Only

on healthcare student knowledge and attitudes. *Age Ageing.* 2020; 49(6):1080-1086. doi:10.1093/ageing/afaa182

24. Fielden AL, Sillence E, Little L. Children's understandings' of obesity, a thematic analysis. *Int J Qual Stud Health Well-Being*. 2011;6.

25. Braun V, Clarke V. Using thematic analysis in psychology. *Qualitative Research in Psychology*. 2006; 3(2):77-101. doi:10.1191/1478088706qp063oa

26. Saunders B, Sim J,Kingstone T, Baker S, Waterfield J, Bartlam B, Jinks C. Saturation in qualitative research: exploring its conceptualization and operationalization. *Qual Quant.* 2018; 52(4):1893-1907. doi:10.1007/s11135-017-0574-8

27. Crane L, Chester JW, Goddard L, Henry LA, Hill E. Experiences of autism diagnosis: A survey of over 1000 parents in the United Kingdom. *Autism.* 2016;20(2):153-62.

28. Liptak GS, Revell, GM. Community physician's role in case management of children with chronic illnesses. *Pediatrics.* 1989; *84*(3):465-471.

29. General Medical Council. *Domain 3: Communication partnership and teamwork.* https://www.gmc-uk.org/ethical-guidance/ethical-guidance-for-doctors/good-medical-practice/domain-3---communication-partnership-and-teamwork [Accessed 1st Jan 2021].

30. National Institue for Health and Care Excellence. *Autism*. https://www.nice.org.uk/guidance/qs51/chapter/quality-statement-3-personalised-plan [Accessed 14th Jan 2021].

31. Carbone PS, Behl DD, Azor V, Murphy NA. The medical home for children with autism spectrum disorders: parent and pediatrician perspectives. *J Autism Dev Disord.* 2010; 40(3):317-324. doi:10.1007/s10803-009-0874-5

32. Levy SE, Frasso R, Colantonio S, Reed H, Stein G, Barg FK, Fiks AG. Shared Decision Making and Treatment Decisions for Young Children With Autism Spectrum Disorder. *Acad Pediatr.* 2016; 16(6):571-578. doi:10.1016/j.acap.2016.04.007

Confidential: For Review Only

33. Royal College of General Practiotioners. *Autism Spectrom Disorders Toolkit.* https://www.rcgp.org.uk/clinical-and-research/resources/toolkits/asd-toolkit.aspx [Accessed 13th Dec 2021].

34. Fiks AG, Localio AR, Alessandrini EA, Asch DA, Guevara JP. Shared decision-making in pediatrics: a national perspective. *Pediatrics.* 2010; 126(2):306-314. doi:10.1542/peds.2010-0526

35. Golnik A, Maccabee-Ryaboy N, Scal P, Wey A, Gaillard P. Shared decision making: improving care for children with autism. *Intellect Dev Disabil.* 2012; 50(4):322-331. doi:10.1352/1934-9556-50.4.322

36. Kuhlthau KA, Bloom S, Van Cleave J, Knapp AA, Romm D, Klatka K, Perrin JM. Evidence for family-centered care for children with special health care needs: a systematic review. *Acad Pediatr.* 2011; 11(2):136-143. doi:10.1016/j.acap.2010.12.014

37. Nicolaidis C, Raymaker DM, Ashkenazy E, McDonald KE, Dern S, Baggs AE, Boisclair WC. "Respect the way I need to communicate with you": Healthcare experiences of adults on the autism spectrum. *Autism.* 2015;19(7):824-831. doi:10.1177/1362361315576221

38. Zuckerman KE, Lindly OJ, Bethell CD, Kuhlthau K. Family impacts among children with autism spectrum disorder: the role of health care quality. *Acad Pediatr.* 2014; 14(4):398-407. doi:10.1016/j.acap.2014.03.011

39. Evans DB, Henschen BL, Poncelet AN, Wilkerson L, Ogur B. Continuity in Undergraduate Medical Education: Mission Not Accomplished. *Journal of general internal medicine.* 2019; 34(10): 2254-2259. doi:10.1007/s11606-019-04949-0

40. Hirsh D, Gaufberg E, Ogur B, Cohen P, Krupat E, Cox M, Bor D. Educational outcomes of the Harvard Medical School-Cambridge integrated clerkship: a way forward for medical education. *Acad Med.* 2012; 87(5): 643-650. doi:10.1097/ACM.0b013e31824d9821

Confidential: For Review Only

41. Ogrinc G, Mutha S, Irby DM. Evidence for longitudinal ambulatory care rotations: a review of the literature. *Acad Med.* 2002; 77(7): 688-693. doi:10.1097/00001888-200207000-00008

42. Magaña S, Parish SL, Rose RA, Timberlake M, Swaine JG. Racial and ethnic disparities in quality of health care among children with autism and other developmental disabilities. *Intellect Dev Disabil.* 2012;50(4): 287-299. doi:10.1352/1934-9556-50.4.287

Confidential: For Review Only

**Table 1 Sociodemographic characteristics**

| Baseline characteristic | n | % | Mean (SD) | Range (years) |
|---|---|---|---|---|
| **Age** | | | 43.64 (7.75) | 29-55 |
| **Age of autistic child.** | | | 10.18 (4.73) | 3-17 |
| **Ethnicity** | | | | |
| White background | 10 | 90.9 | | |
| Mixed background | 1 | 9.1 | | |
| **Relationship to child** | | | | |
| Mother | 10 | 90.9 | | |
| Father | 1 | 9.1 | | |
| **Marital status** | | | | |
| Married/partnered/ cohabiting | 11 | 100 | | |
| **Highest educational level** | | | | |
| No qualification | 1 | 9.1 | | |
| High school/ college | 6 | 54.5 | | |
| University or postgraduate degree | 4 | 36.4 | | |
| **Employment** | | | | |
| Unemployed | 1 | 9.1 | | |
| Employed | 10 | 90.9 | | |

Confidential: For Review Only

**Table 2: Participant quotations**

| Theme/Sub-theme | Quotation |
|---|---|
| **Diagnosis** | *"Dr X who is just amazing, … she knows all about masking and everything…. So, we had the assessment, Dr X listened to what we were saying, and actually listened." (Participant 9)*<br><br>*"This is our GP* [responding to parent concerns]*… "Well, he's not ripping up my surgery, so there can't be much wrong with him" and I just felt like putting my head in my hands, because I just thought … '*<br>*"This is going to be a battle isn't it?" (Participant 1)*<br><br>*"So, again they [the gp] kind of felt …., it was just anxiety, it was kind of anxiety was the kind of prevailing sort of presentation* [and not autism as the parent suspected and was later confirmed] *and … they did the CBT approach." (Participant 7)* |
| **Experiences of doctors** | *"…he [the gp] sort of crouched down…., sort of put his elbows on the bed, and was like really interested in her* [autistic child]. (He) *didn't talk to us* [parents] first*, he talked to her first…So, that was a really good example." (Participant 4)*<br><br>*"…*[autistic child] *wouldn't engage with the doctor, he wouldn't go, so, I rang the doctor, she said "Come and see me" without him* [child]*, to talk about him, which was great, because I was able to go. And she gave me as much time in the world to talk about him and his difficulties and what was going on at school,. And she said, we need to build up his tolerance to come in to visit the doctor. So, she said "Tell you what, next time, make an appointment with me… and if he won't get out the car, I'll come out and see him". (Participant 9)*<br><br>*"the first GP that I saw … I asked at that time for …a referral psychology around his eating, I felt that they just kind of brushed that off and said that "Well, he's too young and it'll kind of sort itself out", but obviously we had several years of quite a restricted eating, So, yeah, so it would have been really good if the GP had kind of spent a bit more time and taken me seriously." (Participant 7)* |
| Parental expectations of doctors | " [medical team]*are relentlessly dismissive and… " (Participant 1)*<br><br>*"'Wwhat would surprise me and make me feel better, is if they were like "Okay, excellent! What's the best way for me to do this with her? Or I need to do this, these are the things I need to do, how's the best way to do it?" (Participant 2)* |
| **TfA considerations**<br><br>Positive attitudes about TfA | *"I think it's brilliant … autism is something that is not well understood … I didn't realise how varied it is and how it displays itself really differently; my child is so, I think it's brilliant to have something that will help health professionals, or doctors in this case, understand a bit more…. So, I think it's great and I really support it." (Participant 2)*<br><br>*"... some of the frustrations that we've experienced really, have been where people have had a very medicalised mechanist understanding of what autism is and … something that is a problem to be fixed,. And so, I think one of the best ways I can think of for new doctors to get a different understanding of it is just to spend time around it, exactly as the programme is proposing. So, I think in that respect that's great." (Participant 6)* |
| Visit considerations | *"... it would just have to make it like a home visit outside of school time, and don't be late. We can't be that flexible, once the time is booked don't change it unless you have to, because that would then probably create some anxiety … but that would just be more* |

Confidential: For Review Only

*because if we've done a lot of work around who's coming* [to the house] *when…" (Participant 4)*

Confidential: For Review Only

### Ethics approval and consent to participate

This study was approved by South Central Hampshire B Research Ethics Committee (Ref 19/SC/0041). Informed written consent was obtained from participants included in the study.

### Consent for publication

Not applicable

### Availability of data and materials

The datasets used and/or analysed during the current study are available from the corresponding author on reasonable request

### Competing interests

The authors declare there are no conflicts of interest.

**Funding**

This study was funded by Health Education England, Kent, Surrey, Sussex

The views expressed are those of the authors and not necessarily those of Health Education England, University of Sussex, University of Brighton or the Department of Health and Social Care

### Author Contributions:

Conception and design: SD, AW, SB, JW

Data collection: SD, LG

Analysis: YD, YF, SD

Manuscript drafting: YD, SD, YF

All authors read and approved the final manuscript.

Confidential: For Review Only

**Acknowledgements**

We would like to thank the parents who took part in this study. We would also like to thank Alison Smith and Dr Lisa Butterworth-Salmon for reviewing the interview topic guides.

Confidential: For Review Only

BMJ Paediatrics Open

# Developing undergraduate autism education for medical students: a qualitative study



| | |
|---|---|
| Journal: | *BMJ Paediatrics Open* |
| Manuscript ID | bmjpo-2022-001411.R2 |
| Article Type: | Original research |
| Date Submitted by the Author: | 10-Jun-2022 |



| | |
|---|---|
| Complete List of Authors: | Dhuga, Yasmin; Brighton and Sussex Medical School<br>Feeney, Yvonne; Brighton and Sussex Medical School<br>Gallaher , Laura ; University of Sussex School of Media Arts and Humanities<br>White, Ann; Sussex Community NHS Foundation Trust<br>Wright , Juliet; Brighton and Sussex Medical School<br>Banerjee, Sube; University of Plymouth Faculty of Health and Human Sciences, Faculty of Health<br>Daley, Stephanie; Brighton and Sussex Medical School, |



| | |
|---|---|
| Keywords: | Health services research, Qualitative research |
| | |


SCHOLARONE™
Manuscripts

*I, the Submitting Author has the right to grant and does grant on behalf of all authors of the Work (as defined in the below author licence), an exclusive licence and/or a non-exclusive licence for contributions from authors who are: i) UK Crown employees; ii) where BMJ has agreed a CC-BY licence shall apply, and/or iii) in accordance with the terms applicable for US Federal Government officers or employees acting as part of their official duties; on a worldwide, perpetual, irrevocable, royalty-free basis to BMJ Publishing Group Ltd ("BMJ") its licensees and where the relevant Journal is co-owned by BMJ to the co-owners of the Journal, to publish the Work in this journal and any other BMJ products and to exploit all rights, as set out in our licence.*

*The Submitting Author accepts and understands that any supply made under these terms is made by BMJ to the Submitting Author unless you are acting as an employee on behalf of your employer or a postgraduate student of an affiliated institution which is paying any applicable article publishing charge ("APC") for Open Access articles. Where the Submitting Author wishes to make the Work available on an Open Access basis (and intends to pay the relevant APC), the terms of reuse of such Open Access shall be governed by a Creative Commons licence – details of these licences and which Creative Commons licence will apply to this Work are set out in our licence referred to above.*

*Other than as permitted in any relevant BMJ Author's Self Archiving Policies, I confirm this Work has not been accepted for publication elsewhere, is not being considered for publication elsewhere and does not duplicate material already published. I confirm all authors consent to publication of this Work and authorise the granting of this licence.*

Confidential: For Review Only

# Developing undergraduate autism education for medical students: a qualitative study

Yasmin Dhuga[a]

Yvonne Feeney[a]

Laura Gallaher[b]

Dr Ann White[c]

Professor Juliet Wright[a]

Professor Sube Banerjee[d]

Dr Stephanie Daley[a]

**Affiliations:**

a. Brighton and Sussex Medical School, UK
b. University of Sussex
c. Sussex Community NHS Foundation Trust, UK
d. Faculty of Health, University of Plymouth, UK

**Corresponding Author**

Dr Stephanie Daley

Address: Brighton & Sussex Medical School, Centre for Dementia Studies, Trafford Building, Room 101, University of Sussex, Falmer, BN1 9RY.

**Email:** s.daley@bsms.ac.uk **Tel:** 00447393 761947 **Twitter handle:** @stephdaleycds

**Word count: 2944**

Confidential: For Review Only

**Abstract**

**Background**:

Autistic adults and children experience considerable health inequalities and have high rates of premature mortality, hospital admissions, and emergency department visits. This is in part due to a lack of autism awareness in the healthcare and social care workforce. A new educational programme, Time for Autism (TfA), for medical students is being developed to address this challenge. This qualitative study was undertaken to support the development of the new programme, in order to a) understand the medical care experiences of parents of autistic children and b) assess their views on the acceptability of the new TfA programme, and willingness to be involved.

**Methods**:

A convenience sample of 11 parents of autistic children were recruited across the South of England. The ages of the autistic children ranged from 3-17 years. Semi-structured interviews were completed between October and December 2019. Interview transcripts were analysed using thematic analysis.

**Results:**

Three key themes were identified: Diagnosis; Experiences of doctors and TfA considerations. There was support for, and willingness to take part in a dedicated autism education programme for medical students, and constructive feedback to inform and improve its delivery.

**Conclusion:** The findings from this study provide insights into the medical care experiences of parents/carers of autistic children. Understanding how parents/carers of autistic children would like medical care to be improved can be used to develop TfA and other autism programmes. Parental/carer support for the development of, and involvement in an autism medical education programme enhances the feasibility of the new programme.

Confidential: For Review Only

*Keywords:* Autism, Medical Education, Qualitative, Healthcare experiences,

Confidential: For Review Only

**Background**

Autism is a multi-faceted condition affecting neurodevelopment. It is characterised by impaired social interaction and communication, restricted interests, and repetitive, stereotypical behaviours (1). There are over 700,000 autistic people in the UK (2), with prevalence increasing (3). The cause of increasing prevalence is unknown but likely to be due in part to increased community awareness amongst the general population, including families and teachers and progress in case identification and definition (4).

Autistic people, both adults and children are more likely to have additional health conditions compared to the general population (5), including both mental health problems such as anxiety-related disorders (6), and sleep disorders (7), as well as physical health problems such as gastrointestinal conditions (8). Autistic adults and children are also at greater risk of health inequalities, and many autistic adults report negative healthcare experiences (9,10). Parents of autistic children describe the healthcare management of their children as inadequate (11). It is widely recognised that poor care experiences, as well as serious concerns about the quality of care provided to autistic adults and children demonstrates a need for improved understanding of autism across the lifespan within the health and social care workforce (12-16).

Medical doctors are expected to have appropriate skills, competencies, and understanding to deliver high quality care to autistic adults and children (14) In England, a competence framework for working with autistic people across the life span has been developed and embedded within statute for the exsiting health and social care workforce(17). However, at the undergraduate medical curriculum level, a time when attitudes and openness to learning can be most malleable, autism teaching provision is often patchy and inconsistent. To enable the future generation of doctors to fully meet the needs of autistic adults and children and deliver care in a way that is person-centred, autism education needs to be routinely embedded in the undergraduate medical curricula. (12,18). There is a gap in the literature

Confidential: For Review Only

about the efficacy of successful interventions that can enhance student understanding of autism.

While the literature on autism education for medical students is limited, there is some evidence indicating that interventions incorporating lived experience can have a positive impact. For example, one study reported an improvement in knowledge and understanding in third year medical students that completed panel discussions with autistic people (19). In another study, the use of a 'digistory' to share the experiences of parents/carers of autistic children led to medical students reporting greater empathy, improved confidence, and an ability to challenge negative stereotypes towards autism (20).

The incorporation of lived experience to raise awareness and enhance skills towards autism in undergraduate medical students is a key feature of a new autism education programme, that is being developed at Brighton and Sussex Medical School. The Time for Autism programme (TfA) will form a mandatory component of the undergraduate medical curriculum. The programme is modelled on a similar educational programme (22) that has successfully enhanced understanding and attitudes of dementia in large cohorts of healthcare students (22,23). In TfA, pairs of medical students will visit a family (parent/carer with an autistic child) on three occasions over one academic year. Students will learn about the lived experience of autism in the context of authentic relationship built between the family and student learners. It is intended that the longitudinal nature of the programme will allow students sufficient time to develop a fuller understanding of how autism affects the child and family over time, further facilitating improved understanding. Supporting teaching activities will focus on autism across the lifespan not just in childhood, and it is anticipated that several of the parents taking part in the new programme, will also have a diagnosis of autism, further enhancing the learning for students.

Confidential: For Review Only

Critical to the establishment of TfA will be the recruitment of volunteer families willing to have medical students visit them at home for the purpose of the new programme. To assess feasibility and inform the development of TfA, a qualitative study was carried out with parent/carers of autistic children to a) understand their experiences of medical care towards their autistic child and b) understand their views about the acceptability, value and potential willingness to get involved in the TfA.

**Methods**

**Study design**

We carried out a qualitative study using individual semi-structured interviews, to explore namely parent/carer views about the medical care experiences of their autistic children as well as a new medical education programme in more depth.

The study considered the *reality* of participants' perceptions through an exploration of their experiences and interpretations they attach to them, within a broader social *construct* (24). This epistemological approach loaned itself to the phenomenological methodology of thematic analysis and is steered by Braun and Clarke's guided approach to thematic analysis (25)

**Setting and participants**

A convenience sample of participants were recruited.  Eligible participants were parents/carers of an autistic child under the age of 18 years living in the South of England. Participants were recruited from local autism charities, local NHS trusts, and online parent forums and they could self-refer to participate in the study.  Participants were approached by email by the researchers and were not known to the research team before interview. Written, informed consent was obtained from all participants.

Confidential: For Review Only

Eleven participants took part in this study. Sociodemographic characteristics are shown in Table 1.

**Insert Table 1 here**

Confidential: For Review Only

There was no specific lived experience advisory involvement in this study, in relation to the study design, delivery and analysis, although this has since been established for the TfA programme.

**Procedure**

Interviews were carried out by two researchers, SD and LG. SD is an experienced qualitative post-doctoral researcher who trained and supervised LG. Each interview was completed at a time and venue suitable to participants; the majority took place at home with only the participant present. Interviews were undertaken on one occasion and were audio recorded and lasted between 30 and 60 minutes. A topic guide was reviewed by two autism clinicians to ensure suitability and interview questions focussed on the experience of the diagnosis of autism, experiences of consulting doctors (generally) about their autistic child, views about the TfA programme, and willingness to participate. A pilot interview did not take place. Transcripts were not returned to participants, and feedback on findings was not sought. At the time of undertaking this study, the TfA programme was at an early stage of its development and we did not have an established lived experience advisory group to call upon to help us to interpret the interview findings.

**Data analysis**

Interviews were transcribed verbatim and checked for accuracy. Transcripts were anonymised, and identifiable data was removed to maintain confidentiality. Thematic saturation was achieved after eleven interviews were completed, therefore data collection stopped at this point (26).

Transcripts were analysed using inductive thematic analysis (25). Familiarity with the data was achieved by reading the transcripts multiple times. To ensure rigour, two separate researchers (YD and YF) independently coded two transcripts, before meeting to discuss the initial codes and resolve disagreements. Fieldwork diaries were also reviewed. The remaining transcripts were independently coded by the lead author; adding more codes when further meaningful data arose. The two researchers worked together to identify relationships between the codes and establish the main themes from the data. The themes were reviewed then refined and appropriate names were assigned to each theme. Microsoft Excel was used to aid the collation of the data.

To enhance the analysis of data, researchers kept fieldwork diaries and made notes after each interview. In addressing reflexivity, the researchers met regularly to discuss their positionality and its impact on the findings during data collection and analysis.

**Ethical considerations**

This study was approved by South Central Hampshire B Research Ethics Committee (Ref 19/SC/0041).

**Results**

Three main themes were identified from the data: Diagnosis, Experiences of doctors, and TfA considerations. These themes are summarised in table 2 and discussed below.

Confidential: For Review Only

**Insert Table 2 here**

**Diagnosis**

Participants reported that the diagnosis of autism was usually provided by either the child's GP or their Paediatrician. The age of diagnosis ranged from 3 to 15 years. This theme captured both positive and negative experiences reported by the participants during the diagnosis of their child.

Positive experiences of diagnosis were influenced by doctors that were non-judgemental, and empathetic about the challenges which participants faced prior to diagnosis. Participants that felt listened to by medical professionals reported better experiences during the diagnosis of their child.

However, participants reported more negative than positive experiences of doctors relating to the diagnosis of autism in their child. The delay in obtaining a diagnosis, and the process of obtaining a diagnosis was often problematic for participants. Several explanations were provided to account for this; including a) a perceived lack of autism awareness b) a perception that parents' concerns were not being taken seriously, and c) perceived difficulty by GPs in identifying symptoms of autism. The presence of comorbid symptoms were perceived to contribute to the challenges obtaining a diagnosis, for example, anxiety was not always viewed as an autistic trait. Another recurrent problem reported was a belief that GP's did not always use the correct diagnostic methods, for example not using standardised tools to enable them to screen for, or to diagnose autism, which delayed diagnosis further.

**Experiences of doctors**

Participants discussed the general experiences they encountered with the wider range of doctors treating their autistic child.

Confidential: For Review Only

Positive experience related to doctors who took a holistic approach and demonstrated patience, and allowed the autistic child time to adapt to new environments. Using personalised approaches and engaging with the child's interests before discussing their wider health issues was appreciated. Participants valued doctors who recognised that autistic children have different needs, and those that took their time to listen and acknowledge parents/carers and their concerns.

Equally, participants discussed many negative experiences of doctors. Most commonly, this related to a perceived lack of autism awareness and experience in doctors.

Some participants described a perceived arrogance in doctors, and a failure to listen to the parent/carer autism-related concerns about their child. As a result, participants suggested that doctors did not adjust their practice to meet the needs of their child causing additional stress for the parent/carers. Some participants felt that GP’s in particular, dismissed their concerns when their children become physically unwell, resulting in avoidance of primary care by parents/carers and use of use of A & E as a first step in receiving medical care. Overall, participants concluded that doctors see autism as a ‘problem’.

Participants expected that all doctors should have an appropriate understanding about autism and a willingness to involve parent/carers in decision making about their autistic child’s care. Participants felt that doctors should involve parents/carers, early in the consultation so they can guide the doctor on how best to lead the consultation. Participants highlighted that as autism manifests differently, doctors should find out what works for each individual autistic child by asking the person who knows them best; their parent. Practical suggestions included doctors physically lowering themselves to the child’s level and the use of easily understood language, rather than jargon.

Confidential: For Review Only

Participants also suggested that doctors should be willing to deviate from routine established practice when assessing an autistic child, because they may present atypically or the child may be unwilling to engage, for example be willing to assess the child in the family car if the child won't come into the GP practice.

**TfA considerations**

This theme focused on participant's views about the TfA programme, and potential willingness to take part. Two subthemes were identified: Positive attitudes about TfA and Visit considerations.

*Positive attitudes about TfA*

All participants were positive about the development of the TfA programme and the potential for it to address the perceived lack of autism awareness in doctors. Participants stated that longitudinal contact with children in the community would enhance medical student understanding of autism and thereby improve their future practice. Participants suggested that gaining direct experience with autistic children would be more valuable than learning about autism from a textbook. Participants also suggested that medical students would be able to learn more about the impact of autism on the family; potentially enabling doctors to better manage difficult family situations. Finally, all participants reported their autistic children would also enjoy being part of TfA.

*Visit considerations*

All participants were keen for TfA to take place and provided practical recommendations to support programme development. Participants advised that students should avoid cancelling or rearranging visits as this could cause anxiety for the child. Participants suggested the need for flexibility during the visits based on the needs of the child, for example, some children might not participate in the conversation while others might enjoy

Confidential: For Review Only

engaging in an activity like drawing pictures. It was perceived that advance information and a photograph of the medical student would reduce possible anxiety in the autistic child.

**Discussion**

This study has generated insights on the medical experiences of families with autism. The results of this qualitative study will be used to inform the development of the TfA programme and can be used to inform other such programmes.

Whilst some of the direct examples provided by participants related to GP's and Paediatricians, participants perceived that most doctors lack awareness of autism and did not work in partnership with parents of autistic children. Participants were strongly supportive of tailored education to address these concerns. Crucially for the development of TfA, participants in this study reported a willingness to take part directly in medical education and share their lived experience to address this challenge. This suggests that the TfA programme is a feasible aspiration.

Participants in in this study highlighted delays in obtaining an autism diagnosis for their child and frustration with the process. A delay of more than 4.5 years between parents detecting their children's developmental issues and receiving a formal autism diagnosis is reported in the literature, and the extent of this diagnostic delay is a predictor of overall parent satisfaction with the diagnostic experience (27). Despite examples of positive experiences, participants overwhelmingly reported negative experiences relating to diagnosis; and perceived that a lack of autism awareness led to delays in diagnosis.

Participants in this study identified that medical management of co-morbidities was often problematic for both parents/carers and the autistic children, leading to differing perceptions of health needs and avoidance of primary care by some participants. This is supported in the

Confidential: For Review Only

wider literature, where it is recognised that co-morbidities in autistic children are often misdiagnosed and mismanaged (28- 30).

Mirroring the wider literature, participants felt they were not regarded as experts in dealing with their children's autism or valued when making joint decisions with doctors (31, 32). These findings demonstrate that doctors must work in partnership with patients/carers of autistic children to improve treatment outcomes (29, 30, 33), as well as improved parent/carer satisfaction.

The success of the doctor's interactions with autistic people depends on their ability to adapt their behaviour to address the needs of the person (37). Unsurprisingly participants in this study valued doctors personalising their approach when communicating with autistic children. However, in order for doctors to adapt their behaviour, they need to be equipped with the necessary attitudes, skills, and knowledge to support autistic people (38).

Participants perceived that TfA would improve student understanding of and approaches towards autistic people. This is supported by research which suggests that this kind of 'real life' exposure enables students to gain positive attitudes and values that cannot be learnt through textbooks (39). Longitudinal educational models that offer continuity of contact between student learners and patients/carers can enhance insight into the psychosocial side of caring for patients and understanding of chronic health conditions (40,41).

This study has three main limitations. First, this study used a convenience sample, and therefore lacks diversity in terms of ethnicity and economic status. Our participants were predominantly white, female parents/carers. We were therefore not able to assess the experiences of all groups of carers, which is of concern as it is suggested that autistic children from ethnic backgrounds experience more barriers in the healthcare setting (42). Additionally, it is not clear whether parents from more diverse backgrounds would be willing

Confidential: For Review Only

to take part in a programme like TfA, potentially limiting exposure to the full range of experiences for our medical students. It is evident that further research using a more diverse sample of participants is necessary. Second, this study was undertaken in the south of England, and given the variation in support available geographically for autistic children (27), further research across a wider geographic area would be beneficial. Finally, lived experience involvement of parents/carers to advise on the design, delivery and analysis would have strengthened the study.

A strength of this study is an improved understanding of the medical care experiences of autistic children and their parents/carers for the purposes of the design and delivery of autism education in undergraduate healthcare curricula.  Their positive attitudes towards TfA demonstrates enthusiasm for the active involvement of parents/carers and autistic children in educational delivery.

## Conclusion

Despite calls for improved autism knowledge and awareness in the medical profession, this study found that parents/carers of autistic children perceived a generalised lack of understanding of autism in medical settings.

While there is a lack of evidence demonstrating new and innovative ways to enhance autism awareness, education that incorporates lived experience can enhance positive attitudes and values towards chronic conditions.  Findings from this study can be used in the design of new initiatives to improve autism awareness in healthcare education. Participants in this study were willing to take part in new programmes, such as TfA to improve understanding of autism in future doctors. A full evaluation of the experience of medical students and parents/carers in TfA will be undertaken on the new programme.

Confidential: For Review Only

**What is already known on this topic**

- Parents/children of autistic children have high levels of dissatisfaction with the medical care provided to their children
- There is a lack of autism knowledge in doctors which contributes towards this problem
- For medical students, there is a lack of evidence as to how this knowledge gap can be addressed

**What this study adds**

- The identification of areas of practice in which parents/carers perceive that autism education should be addressed in medical students
- The willingness of parents/carers to contribute their lived experience within in autism education to address these concerns
- The introduction of a new autism education model, Time for Autism, for medical students in which lived experience is a core component

**Acknowledgements**

We would like to thank the parents who took part in this study. We would also like to thank Alison Smith and Dr Lisa Butterworth-Salmon for reviewing the interview topic guides.

Confidential: For Review Only

## References


1. American Psychiatric Association. *Diagnostic and Statistical Manual of Mental Disorders. 5th ed. (DSM-5)*, 2013 Washington, DC: American Psychiatric Association.
2. National Autistic Society. *What is autism?* https://www.autism.org.uk/advice-and-guidance/what-is-autism [Accessed 3rd Dec 2020].
3. King M, Bearman P, Diagnostic change and the increased prevalence of autism. *International Journal of Epidemiology*. 2009; 38(5): 1224–1234. doi.org/10.1093/ije/dyp261
4. Zeidan J, Fombonne E, Scorah J, Ibrahim A, Durkin MS, Saxena S, Yusuf A, Shih A, & Elsabbagh, M. Global prevalence of autism: A systematic review update. *Autism Research*. 2022; 1– 13. doi.org/10.1002/aur.2696
5. Saqr Y, Braun E, Porter K, Barnette D, Hanks C. Addressing medical needs of adolescents and adults with autism spectrum disorders in a primary care setting. *Autism.* 2017; *22*(1):51-61. doi:10.1177/1362361317709970
6. van Steensel FJ, Bögels SM, Perrin S. Anxiety disorders in children and adolescents with autistic spectrum disorders: a meta-analysis. *Clin Child Fam Psychol Rev.* 2011;14(3):302-317. doi:10.1007/s10567-011-0097-0
7. Kotagal S, Broomall E. Sleep in Children With Autism Spectrum Disorder. *Pediatric Neurology.* 2012; *47*(4):242-251. doi:https://doi.org/10.1016/j.pediatrneurol.2012.05.007
8. Chaidez V, Hansen RL, Hertz-Picciotto I. Gastrointestinal problems in children with autism, developmental delays or typical development. *J Autism Dev Disord*. 2014; *44*(5):1117-1127. doi:10.1007/s10803-013-1973-x.
9. Nicolaidis C, Raymaker D, McDonald K, Dern S, Boisclair WC, Ashkenazy E, Baggs A. Comparison of healthcare experiences in autistic and non-autistic adults: a cross-sectional online survey facilitated by an academic-community partnership. *J Gen Intern Med.* 2013; *28*(6):761-769. doi:10.1007/s11606-012-2262-7.


Confidential: For Review Only

10. Liptak GS, Orlando M, Yingling JT, Theurer-Kaufman KL, Malay DP, Tompkins LA, Flynn JR. Satisfaction with primary health care received by families of children with developmental disabilities. *J Pediatr Health Care.* 2006; 20(4):245-252. doi:10.1016/j.pedhc.2005.12.008

11. Nik Adib NA, Ibrahim MI, Ab Rahman A, Bakar RS, Yahaya NA, Hussin S, Wan Mansor WNA. Predictors of Caregivers' Satisfaction with the Management of Children with Autism Spectrum Disorder: A Study at Multiple Levels of Health Care. *Int J Environ Res Public Health*. 2019; 16(10). doi:10.3390/ijerph16101684

12. Austriaco K, Aban I, Willig J, Kong M. Contemporary Trainee Knowledge of Autism: How Prepared Are Our Future Providers? *Front Pediatr.* 2019; *7*:165. doi:10.3389/fped.2019.00165

13. Bruder MB, Kerins G, Mazzarella C, Sims J, Stein N. Brief report: the medical care of adults with autism spectrum disorders: identifying the needs. *J Autism Dev Disord.* 2012;42(11):2498-2504. doi:10.1007/s10803-012-1496-x

14. Care Quality Commission. Out of sight – who cares?: Restraint, segregation and seclusion review. https://www.cqc.org.uk/sites/default/files/20201218_rssreview_report.pdf [Accessed 3rd Dec 2020].

15. Drum C, Krahn G, Peterson-Besse J, Horner-Johnson W, Newton K. Health of people with disabilities: Determinants and disparities. *Disability and Public Health*. 2009; 125-144.

16. World Health Organisation, 2022, Autism, World Health Organisation- Geneva (report). https://www.who.int/news-room/fact-sheets/detail/autism-spectrum-disorders

17. Department of Health & Social Care. *Core Capabilities Framework for Supporting Autistic People.* [Internet] 2019. https://skillsforhealth.org.uk/wp-content/uploads/2020/11/Autism-Capabilities-Framework-Oct-2019.pdf. Accessed 2021 January 15].

Confidential: For Review Only

18. Ellias SD, Shah HR. A Study of Assessment of Knowledge of Childhood Autism among Medical Students in Mumbai. *Ann Indian Acad Neuro.* 2019; 22(2):164-169. doi:10.4103/aian.AIAN_486_17.

19. Havercamp SM, Ratliff-Schaub K, Macho PN, Johnson CN, Bush KL, Souders HT. Preparing tomorrow's doctors to care for patients with autism spectrum disorder. *Intellectual and Developmental Disabilities*. 2016; 54(3):202-216.

20. Codd A, Burford B, Petruso G, Davidson N, Vance G. Development and evaluation of a digistory about autistic spectrum disorder - a pilot study. *Educ Prim Care.* 2018;29(4):232-236. doi:10.1080/14739879.2018.1470906

21. Banerjee S, Farina N, Daley S, Grosvenor W, Hughes L, Hebditch M, Wright J. How do we enhance undergraduate healthcare education in dementia? A review of the role of innovative approaches and development of the Time for Dementia Programme. *Int J Geriatr Psychiatry.* 2016; *32*(1):68-75.

22. Banerjee S, Jones C, Wright J, Grosvenor W, Hebditch M, Hughes L, Daley S. A comparative study of the effect of the Time for Dementia programme on medical students. *Int J Geriatr Psychiatry.* 2021; 36(7):1011-1019. doi:10.1002/gps.5532

23. Daley S, Feeney Y, Grosvenor W, Hebditch M, Morley L, Sleater G, Banerjee S. A qualitative evaluation of the effect of a longitudinal dementia education programme on healthcare student knowledge and attitudes. *Age Ageing.* 2020; 49(6):1080-1086. doi:10.1093/ageing/afaa182

24. Fielden AL, Sillence E, Little L. Children's understandings' of obesity, a thematic analysis. *Int J Qual Stud Health Well-Being*. 2011;6.

25. Braun V, Clarke V. Using thematic analysis in psychology. *Qualitative Research in Psychology*. 2006; 3(2):77-101. doi:10.1191/1478088706qp063oa

26. Saunders B, Sim J,Kingstone T, Baker S, Waterfield J, Bartlam B, Jinks C. Saturation in qualitative research: exploring its conceptualization and operationalization. *Qual Quant.* 2018; 52(4):1893-1907. doi:10.1007/s11135-017-0574-8

Confidential: For Review Only

27. Crane L, Chester JW, Goddard L, Henry LA, Hill E. Experiences of autism diagnosis: A survey of over 1000 parents in the United Kingdom. *Autism.* 2016;20(2):153-62.

28. Liptak GS, Revell, GM. Community physician's role in case management of children with chronic illnesses. *Pediatrics.* 1989; *84*(3):465-471.

29. General Medical Council. *Domain 3: Communication partnership and teamwork.* https://www.gmc-uk.org/ethical-guidance/ethical-guidance-for-doctors/good-medical-practice/domain-3---communication-partnership-and-teamwork [Accessed 1st Jan 2021].

30. National Institue for Health and Care Excellence. *Autism*. https://www.nice.org.uk/guidance/qs51/chapter/quality-statement-3-personalised-plan [Accessed 14th Jan 2021].

31. Carbone PS, Behl DD, Azor V, Murphy NA. The medical home for children with autism spectrum disorders: parent and pediatrician perspectives. *J Autism Dev Disord.* 2010; 40(3):317-324. doi:10.1007/s10803-009-0874-5

32. Levy SE, Frasso R, Colantonio S, Reed H, Stein G, Barg FK, Fiks AG. Shared Decision Making and Treatment Decisions for Young Children With Autism Spectrum Disorder. *Acad Pediatr.* 2016; 16(6):571-578. doi:10.1016/j.acap.2016.04.007

33. Royal College of General Practiotioners. *Autism Spectrom Disorders Toolkit.* https://www.rcgp.org.uk/clinical-and-research/resources/toolkits/asd-toolkit.aspx [Accessed 13th Dec 2021].

34. Fiks AG, Localio AR, Alessandrini EA, Asch DA, Guevara JP. Shared decision-making in pediatrics: a national perspective. *Pediatrics.* 2010; 126(2):306-314. doi:10.1542/peds.2010-0526

35. Golnik A, Maccabee-Ryaboy N, Scal P, Wey A, Gaillard P. Shared decision making: improving care for children with autism. *Intellect Dev Disabil.* 2012; 50(4):322-331. doi:10.1352/1934-9556-50.4.322

36. Kuhlthau KA, Bloom S, Van Cleave J, Knapp AA, Romm D, Klatka K, Perrin JM. Evidence for family-centered care for children with special health care needs: a

Confidential: For Review Only

systematic review. *Acad Pediatr.* 2011; 11(2):136-143. doi:10.1016/j.acap.2010.12.014

37. Nicolaidis C, Raymaker DM, Ashkenazy E, McDonald KE, Dern S, Baggs AE, Boisclair WC. "Respect the way I need to communicate with you": Healthcare experiences of adults on the autism spectrum. *Autism.* 2015;19(7):824-831. doi:10.1177/1362361315576221
38. Zuckerman KE, Lindly OJ, Bethell CD, Kuhlthau K. Family impacts among children with autism spectrum disorder: the role of health care quality. *Acad Pediatr.* 2014; 14(4):398-407. doi:10.1016/j.acap.2014.03.011
39. Evans DB, Henschen BL, Poncelet AN, Wilkerson L, Ogur B. Continuity in Undergraduate Medical Education: Mission Not Accomplished. *Journal of general internal medicine.* 2019; 34(10): 2254-2259. doi:10.1007/s11606-019-04949-0
40. Hirsh D, Gaufberg E, Ogur B, Cohen P, Krupat E, Cox M, Bor D. Educational outcomes of the Harvard Medical School-Cambridge integrated clerkship: a way forward for medical education. *Acad Med.* 2012; 87(5): 643-650. doi:10.1097/ACM.0b013e31824d9821
41. Ogrinc G, Mutha S, Irby DM. Evidence for longitudinal ambulatory care rotations: a review of the literature. *Acad Med.* 2002; 77(7): 688-693. doi:10.1097/00001888-200207000-00008
42. Magaña S, Parish SL, Rose RA, Timberlake M, Swaine JG. Racial and ethnic disparities in quality of health care among children with autism and other developmental disabilities. *Intellect Dev Disabil.* 2012;50(4): 287-299. doi:10.1352/1934-9556-50.4.287

Confidential: For Review Only

**Table 1 Sociodemographic characteristics**

| Baseline characteristic | n | % | Mean (SD) | Range (years) |
|---|---|---|---|---|
| **Age** | | | 43.64 (7.75) | 29-55 |
| **Age of autistic child.** | | | 10.18 (4.73) | 3-17 |
| **Ethnicity** | | | | |
| White background | 10 | 90.9 | | |
| Mixed background | 1 | 9.1 | | |
| **Relationship to child** | | | | |
| Mother | 10 | 90.9 | | |
| Father | 1 | 9.1 | | |
| **Marital status** | | | | |
| Married/partnered/ cohabiting | 11 | 100 | | |
| **Highest educational level** | | | | |
| No qualification | 1 | 9.1 | | |
| High school/ college | 6 | 54.5 | | |
| University or postgraduate degree | 4 | 36.4 | | |
| **Employment** | | | | |
| Unemployed | 1 | 9.1 | | |
| Employed | 10 | 90.9 | | |

Confidential: For Review Only

**Table 2: Participant quotations**

| Theme/Sub-theme | Quotation |
|---|---|
| **Diagnosis** | *"Dr X who is just amazing, … she knows all about masking and everything…. So, we had the assessment, Dr X listened to what we were saying, and actually listened." (Participant 9)*<br><br>*"This is our GP* [responding to parent concerns]… *"Well, he's not ripping up my surgery, so there can't be much wrong with him" and I just felt like putting my head in my hands, because I just thought … '*<br>*"This is going to be a battle isn't it?" (Participant 1)*<br><br>*"So, again they [the gp] kind of felt …., it was just anxiety, it was kind of anxiety was the kind of prevailing sort of presentation* [and not autism as the parent suspected and was later confirmed] *and … they did the CBT approach." (Participant 7)* |
| **Experiences of doctors** | *"…he [the gp] sort of crouched down…., sort of put his elbows on the bed, and was like really interested in her* [autistic child]. (He) *didn't talk to us* [parents] first, *he talked to her first…So, that was a really good example." (Participant 4)*<br><br>*"…*[autistic child] *wouldn't engage with the doctor, he wouldn't go, so, I rang the doctor, she said "Come and see me" without him* [child], *to talk about him, which was great, because I was able to go. And she gave me as much time in the world to talk about him and his difficulties and what was going on at school,. And she said, we need to build up his tolerance to come in to visit the doctor. So, she said "Tell you what, next time, make an appointment with me… and if he won't get out the car, I'll come out and see him". (Participant 9)*<br><br>*"the first GP that I saw … I asked at that time for …a referral psychology around his eating, I felt that they just kind of brushed that off and said that "Well, he's too young and it'll kind of sort itself out", but obviously we had several years of quite a restricted eating, So, yeah, so it would have been really good if the GP had kind of spent a bit more time and taken me seriously." (Participant 7)* |
| Parental expectations of doctors | *"* [medical team]*are relentlessly dismissive and… " (Participant 1)*<br><br>*"'Wwhat would surprise me and make me feel better, is if they were like "Okay, excellent! What's the best way for me to do this with her? Or I need to do this, these are the things I need to do, how's the best way to do it?" (Participant 2)* |
| **TfA considerations**<br><br>Positive attitudes about TfA | *"I think it's brilliant … autism is something that is not well understood … I didn't realise how varied it is and how it displays itself really differently; my child is so, I think it's brilliant to have something that will help health professionals, or doctors in this case, understand a bit more…. So, I think it's great and I really support it." (Participant 2)*<br><br>*"... some of the frustrations that we've experienced really, have been where people have had a very medicalised mechanist understanding of what autism is and … something that is a problem to be fixed,. And so, I think one of the best ways I can think of for new doctors to get a different understanding of it is just to spend time around it, exactly as the programme is proposing. So, I think in that respect that's great." (Participant 6)* |
| Visit considerations | *"... it would just have to make it like a home visit outside of school time, and don't be late. We can't be that flexible, once the time is booked don't change it unless you have to, because that would then probably create some anxiety … but that would just be more* |

Confidential: For Review Only

*because if we've done a lot of work around who's coming* [to the house] *when…" (Participant 4)*

Confidential: For Review Only

**Ethics approval and consent to participate**

This study was approved by South Central Hampshire B Research Ethics Committee (Ref 19/SC/0041). Informed written consent was obtained from participants included in the study.

**Consent for publication**

Not applicable

**Availability of data and materials**

The datasets used and/or analysed during the current study are available from the corresponding author on reasonable request

**Competing interests**

The authors declare there are no conflicts of interest.

**Funding**

This study was funded by Health Education England, Kent, Surrey, Sussex

The views expressed are those of the authors and not necessarily those of Health Education England, University of Sussex, University of Brighton or the Department of Health and Social Care

**Author Contributions:**

Conception and design: SD, AW, SB, JW

Data collection: SD, LG

Analysis: YD, YF, SD

Manuscript drafting: YD, SD, YF

All authors read and approved the final manuscript.

Confidential: For Review Only

**Acknowledgements**

We would like to thank the parents who took part in this study. We would also like to thank Alison Smith and Dr Lisa Butterworth-Salmon for reviewing the interview topic guides.

Confidential: For Review Only
